# Influence of Surfactant-Assisted Graphene Nanoplatelet Dispersion on the Mechanical and Electrical Properties of Fly Ash Geopolymer Mortar

**DOI:** 10.3390/ma19194098

**Published:** 2026-09-25

**Authors:** Mahmoud Shakouri, Mohammad Wais Sulaimani

**Affiliations:** 1 Department of Construction Management, Boise State University, Boise, ID 83725, USA; 2 Department of Construction Management, Colorado State University, Fort Collins, CO 80523, USA; wais.sulaimani@colostate.edu

**Keywords:** fly ash geopolymer mortar, graphene nanoplatelets, surfactant-assisted dispersion, bulk electrical resistivity, desirability analysis

## Abstract

Developing electrically functional geopolymer composites requires conductive fillers that improve electrical response while maintaining mechanical performance. This study examined that formulation tradeoff using graphene nanoplatelets (GNPs) in fly ash geopolymer mortar. An aqueous screening program comprised 48 dispersions prepared with sodium dodecyl sulfate (SDS), Triton X-100, or polyvinylpyrrolidone, combining four GNP concentrations with four dispersant-to-GNP mass ratios. Conductivity, pH, and visual observations over 12 days supported selecting SDS for subsequent mortar preparation, although these measurements did not establish superior nanoscale dispersion or long-term alkaline stability. Subsequent testing evaluated 16 SDS-GNP mortar mixtures alongside four SDS-only reference mixtures. Dispersion concentrations of 0.05, 0.10, 0.50, and 1.00 wt.% corresponded to approximately 0.0043, 0.0085, 0.0426, and 0.0852 wt.% GNP relative to fly ash. Compressive strength ranged from approximately 5.5 to 16.8 MPa, with higher loading generally associated with lower strength. At the lowest SDS-to-GNP ratio, increasing dispersion concentration from 0.10% to 1.00% reduced strength from about 11.8 to 7.2 MPa, a decrease of approximately 39%. Ultrasonic pulse velocity followed a similar trend, reaching about 3.0 km/s at lower concentrations compared with 2.08 to 2.50 km/s for the 1.00% mixtures. Bulk electrical resistivity generally ranged from 60 to 180 Ω·m after ambient storage and from 5 to 29 Ω·m after water immersion, without a consistent decrease as the GNP concentration increased. Factorial analyses indicated formulation interactions, while desirability analysis favored selected low-concentration mixtures for their combined mechanical and electrical responses. Lower GNP-dispersion concentrations therefore provided a more favorable balance under the tested conditions, but incomplete SDS-dose matching prevents attribution of the observed changes to GNPs alone. These results support formulation screening without establishing conductive-network formation or validating sensing and heating applications.

## 1. Introduction

Ordinary portland cement production is associated with substantial CO_2_ emissions from both limestone decarbonation and fuel combustion during clinker production. Although concrete can reabsorb a portion of this CO_2_ through natural carbonation during service, Xiao et al. [1] projected that this uptake would offset less than 10% of annual clinker-related emissions. Carbonation alone therefore cannot compensate for the emissions associated with conventional clinker production. This limitation supports the need to reduce reliance on high-clinker OPC systems and to investigate alternative binder systems, including geopolymer and alkali-activated materials, that can reduce or replace clinker use.

Geopolymer binders have been investigated as alternatives to ordinary portland cement-based materials because they can be produced through alkaline activation of aluminosilicate precursors such as fly ash and metakaolin, with slag or other industrial and mineral sources incorporated in some formulations [2,3,4,5,6]. In fly ash-based systems, dissolution of reactive Si- and Al-bearing phases is followed by the formation of alkali aluminosilicate reaction products. The composition and structure of these products depend on precursor reactivity, activator chemistry, curing conditions, calcium availability, and mixture proportions [2,3,4,5,6,7]. Because physical and chemical properties vary widely among different fly ash sources, bulk oxide composition alone cannot predict the resulting engineering performance [4,7]. Performance must therefore be evaluated for each specific formulation and intended application.

While adequate engineering performance remains a central consideration for conventional geopolymer applications, research has also expanded toward multifunctional composites that combine mechanical performance with electrical, sensing, thermal, energy-storage, or environmental functions [2,8,9,10]. Potential applications include structural health monitoring, smart pavement systems, and electromagnetic shielding. In conventional durability assessment of reinforced concrete, higher resistivity is generally associated with lower ionic conductivity and a reduced likelihood of corrosion activity [11,12]. In electrically responsive mortar, however, lower resistivity may be desirable to facilitate electrical transport or sensing.

To tailor this electrical response, electrically active fillers such as carbon fibers, carbon nanotubes, graphene, graphene oxide, reduced graphene oxide, and graphene nanoplatelets (GNPs) have been investigated [2,9,13,14,15,16,17,18,19,20]. When adequately dispersed and sufficiently connected within the matrix, these fillers can contribute to electrical transport, particle bridging, interfacial modification, and sensing response [9,17]. GNPs are particularly attractive for multifunctional composite applications because of their layered morphology, commercial availability, and potential electrical conductivity [14,16,17,18]. However, the high intrinsic conductivity of GNP powder does not necessarily translate into low composite resistivity. GNPs can reassociate and form stacked structures, which can reduce accessible surface area and produce heterogeneous inclusions within the matrix [21,22,23]. This creates a formulation challenge because improving electrical response through GNP addition can be accompanied by losses in workability or mechanical performance when dispersion is inadequate. Agglomerated particles can create weak regions or stress concentrations, increase liquid demand, and disrupt matrix continuity [7,14,16,20,24,25,26].

Surfactants and polymeric stabilizers are therefore commonly considered to improve particle separation and limit reagglomeration during dispersion. Different classes of dispersing agents act through different stabilization mechanisms. Sodium dodecyl sulfate (SDS), Triton X-100 (TX-100), and polyvinylpyrrolidone (PVP) represent an anionic surfactant, a nonionic surfactant, and a polymeric stabilizer, respectively. SDS can promote electrostatic stabilization, whereas TX-100 and PVP can provide steric stabilization through adsorption at the particle–liquid interface. Nazari et al. [27] compared several surfactants for aqueous GNP dispersion and found that cetyltrimethylammonium bromide (CTAB) provided the highest dispersion stability and smallest flake size. Jiang et al. [22] further showed that GNP dispersion depended on the combined effects of surfactant type, surfactant-to-GNP ratio, and sonication conditions. Chuah et al. [21] demonstrated that the aggregation behavior of graphene oxide in cement-related pore solutions was affected by calcium concentration and alkalinity.

These studies indicate that dispersion stability can change when graphene-based particles are introduced into a chemically complex alkaline binder environment. This sensitivity is relevant to fly ash-based geopolymer mortar, where GNPs and surfactants interact with alkaline activators, dissolved species, precursor particles, and aggregate. These interactions may influence particle dispersion, surfactant behavior, air entrainment, matrix development, and ultimately the mechanical and electrical response of the composite [16,21,22,23,27]. Selection of a suitable GNP–surfactant system therefore requires consideration of both dispersion behavior and the resulting engineering performance.

Previous studies have established the feasibility of incorporating graphene-based materials into geopolymer systems [7,13,16]. The remaining formulation challenge is to determine whether improvements in electrical response can be achieved without unacceptable reductions in fresh-state or hardened performance and how this balance depends on both GNP concentration and surfactant dosage. Because these responses have different units and may favor different mixture conditions, multi-response desirability analysis offers a practical approach for integrating them into a common basis for mixture evaluation [28,29].

The present study evaluates the surfactant-assisted incorporation of GNPs into Class F fly ash-based geopolymer mortar. The objectives are to: (1) screen SDS, TX-100, and PVP as candidate dispersing agents for GNPs; (2) evaluate the effects of GNP concentration, surfactant-to-GNP ratio, and their interaction on workability, compressive strength, ultrasonic pulse velocity (UPV), and bulk electrical resistivity; and (3) use multi-response desirability analysis to identify mixture combinations that provide the most favorable balance between reduced electrical resistivity and maintained fresh-state, mechanical, and ultrasonic performance within the investigated range.

## 2. Materials and Methods

### 2.1. Materials

#### 2.1.1. Fly Ash

In this study, Class F fly ash conforming to ASTM C618 [30] was used as the primary aluminosilicate precursor. Its chemical composition is presented in Table 1. The fly ash had a reported median particle size of 16.34 μm, specific surface area of 598 m^2^/kg, and density of 2.39 g/cm^3^.

#### 2.1.2. Graphene Nanoplatelets

Commercial-grade graphene nanoplatelets were used as the conductive filler in this study. Supplier-reported properties were used for the initial material screening and were supplemented by independent characterization, as detailed in Section 2.2.1. The GNPs had a reported purity greater than 90 wt.%, a particle diameter of 0.5–0.7 μm, and fewer than 15 graphene layers.

#### 2.1.3. Sodium Silicate

A commercial-grade sodium silicate solution was used as the soluble silicate component of the alkaline activator, which also provided a portion of the mixture water. The solution consisted of 28 wt.% ${\mathrm{SiO}}_{2}$, 9 wt.% ${\mathrm{Na}}_{2}O$, and 63 wt.% water. This yielded an ${\mathrm{SiO}}_{2}/{\mathrm{Na}}_{2}O$ mass ratio of 3.11 and a molar ratio of approximately 3.21. The supplier-reported pH was 11.3, with a density of 1.39 g/cm^3^ at 20 °C.

#### 2.1.4. Other Chemicals

Reagent-grade sodium hydroxide flakes with a reported purity greater than 99% were used. The dispersing agents included SDS, TX-100, and PVP. Table 2 summarizes their reported physicochemical properties.

#### 2.1.5. Fine Aggregates

Washed multi-purpose sand was used as the fine aggregate in all mortar mixtures to maintain a consistent aggregate source across the experimental program. The sand had a bulk specific gravity of 2.63, an apparent specific gravity of 2.71, a water absorption of 1.75%, and a fineness modulus of 2.90.

### 2.2. Experimental Program

The experimental program was conducted in three phases. Phase 1 characterized the commercial GNPs to evaluate their platelet morphology, graphitic structure, defect-related features, and surface bonding states before mixture preparation. Phase 2 evaluated the aqueous dispersion behavior of GNP–surfactant systems using 48 formulations prepared in distilled water. Suspension behavior was assessed through visual observations of sedimentation, clustering, and phase separation, supplemented by electrical conductivity and pH measurements. Based on this screening, one surfactant was selected for further evaluation. Phase 3 incorporated the selected surfactant into fly ash-based geopolymer mortar and evaluated the effects of GNP dosage and surfactant-to-GNP mass ratio on workability, compressive strength, ultrasonic pulse velocity, and bulk electrical resistivity under two moisture conditions. Control mixtures containing the selected surfactant without GNPs were included to distinguish surfactant-related effects from those associated with GNP addition.

#### 2.2.1. GNP Characterization

The commercial GNPs were characterized before mixture preparation to verify that the supplied material exhibited morphological, structural, and surface-chemical characteristics consistent with graphene nanoplatelets and to establish a baseline description of the starting material.

The commercial GNPs were characterized before mixture preparation using transmission electron microscopy (TEM), scanning electron microscopy (SEM), Raman spectroscopy, and X-ray photoelectron spectroscopy (XPS). Electron microscopy was used to examine platelet morphology and lateral geometry, Raman spectroscopy evaluated graphitic structure and defect-related features, and XPS assessed near-surface elemental composition and carbon bonding states.

For TEM, approximately 5 mg of dry GNP powder was dispersed in ethanol and ultrasonicated for 10 min. A droplet of the ethanolic dispersion was deposited onto a carbon-coated copper grid and dried before imaging. TEM was performed using a Hitachi HT7700 microscope (Hitachi High-Technologies Corporation, Tokyo, Japan) operated at 120.0 kV, and the representative micrograph was collected at 50,000× magnification.

For SEM, dry GNP powder was mounted on conductive carbon tape attached to an aluminum stub. The powder was gently spread, and loosely attached particles were removed by tapping. No sputter coating was applied because GNPs are electrically conductive, and imaging was conducted at a low accelerating voltage. SEM was performed using a ZEISS Sigma 300 field-emission microscope (Carl Zeiss Microscopy GmbH, Jena, Germany) equipped with an InLens detector. The representative image was collected at 1.00 kV, a working distance of 4.5 mm, and 5000× magnification.

Raman spectroscopy was performed using a WITec alpha300 R Raman microscope equipped with a 532 nm laser (WITec Wissenschaftliche Instrumente und Technologie GmbH, Ulm, Germany). Dry GNP powder was spread on a quartz slide and analyzed at room temperature using a 20× objective, approximately 0.1 mW laser power, 0.5 s integration time, and 200 accumulations. The spectra were smoothed using the Savitzky–Golay method and baseline corrected.

XPS was performed using a Thermo Scientific ESCALAB 250Xi spectrometer (Thermo Fisher Scientific, East Grinstead, UK) equipped with a monochromatic Al Kα source with a photon energy of 1486.6 eV. GNP powder was pressed onto indium foil and analyzed under ultrahigh vacuum. Survey spectra were collected using a pass energy of 150 eV. High-resolution C 1s and O 1s spectra were collected using a pass energy of 20 eV. Binding energies were corrected using the C 1s reference peak at 284.8 to 285.0 eV. The C 1s spectrum was fitted after background subtraction using nonlinear least-squares curve fitting.

#### 2.2.2. Aqueous Suspension Stability Screening

Phase I evaluated the suspension stability of GNP–surfactant systems in water. The screening matrix combined three dispersing agents, SDS, TX-100, and PVP, with GNP concentrations of 0.05, 0.10, 0.50, and 1.00 wt.% relative to the mass of water and surfactant-to-GNP mass ratios of 0.5:1, 1:1, 1.5:1, and 2:1. These levels were selected to span GNP concentrations and dispersant dosages representative of ranges investigated in previous GNP dispersion and cementitious-composite studies [22,27]. The GNP concentrations included two lower levels, 0.05 and 0.10 wt.%, and two higher levels, 0.50 and 1.00 wt.%, with the highest concentration being 20 times the lowest. This range was selected to examine how suspension behavior changed as GNP concentration increased. The surfactant-to-GNP ratios provided surfactant masses below, equal to, and above the GNP mass, allowing the influence of relative surfactant dosage to be compared across the selected GNP concentrations. These concentrations and ratios were used as discrete screening levels rather than as predetermined optimum dosages. Combining the three dispersing agents, four GNP concentrations, and four mass ratios produced 48 aqueous suspensions.

The suspensions were compared using macroscopic stability, electrical conductivity, and pH as practical screening indicators for selecting a GNP–surfactant system for Phase II. Macroscopic observations were used to assess visible sedimentation, separation, and persistence of the suspended GNPs over time. Electrical conductivity provided a complementary measure of the ionic characteristics of the suspension, particularly for systems containing ionic surfactants, while pH was monitored to identify differences in the chemical conditions among the candidate systems. Table 3 summarizes the surfactant mass corresponding to each surfactant-to-GNP ratio.

Each suspension was prepared by first adding the required surfactant mass to distilled water in a 500 mL beaker. The solution was mixed for 5 min at 300 rpm using an OHAUS Guardian 5000 magnetic stirrer (OHAUS Corporation, Parsippany, NJ, USA) to distribute the surfactant before GNP addition. The required GNP mass was then added, and the suspension was mixed for 30 min at 300 rpm using a LabFish overhead stirrer (Zhejiang Huili Experimental Instrument Co., Ltd., Huzhou, China). The suspension was subsequently ultrasonicated at 40 kHz for 1 h while the temperature was maintained below 35 °C.

These preparation conditions were selected based on preliminary trials conducted before the main experimental program. Among the conditions evaluated, the combination of mechanical mixing and ultrasonication provided the most satisfactory macroscopic suspension behavior. The same preparation procedure was therefore applied to all formulations to maintain a consistent basis for comparison. The procedure was not intended to establish an optimized sonication condition because GNP dispersion can also depend on delivered ultrasonic energy, temperature history, particle concentration, and surfactant chemistry [24,31]. Systematic optimization of these parameters was beyond the scope of this study.

Immediately after preparation, electrical conductivity and pH were measured using a SevenCompact pH/conductivity meter (Mettler-Toledo, LLC, Columbus, OH, USA). These measurements were used as complementary indicators of the chemical and ionic characteristics of the GNP–surfactant suspensions rather than as direct measures of GNP dispersion.

Macroscopic suspension stability was evaluated visually. Preliminary UV–Vis measurements of the undiluted suspensions produced excessive absorbance and light scattering, preventing reliable quantitative comparison under the conditions tested. Dilution could have brought the measurements within the instrument range, but it would also have changed the absolute GNP and surfactant concentrations and could have altered adsorption and colloidal interactions, even though the surfactant-to-GNP mass ratio would remain unchanged. Because a validated dilution and UV–Vis measurement protocol was not established, visual assessment was used as a qualitative indicator of macroscopic suspension stability rather than as a quantitative measure of dispersed GNP concentration.

Following preparation, the suspensions were transferred to sealed vials and stored undisturbed for 12 d. At the end of the storage period, each suspension was examined for visible sedimentation, particle clusters, and phase separation.

#### 2.2.3. Mortar Mixture Design Matrix

The mortar mixtures were prepared using the surfactant selected from the preliminary dispersion-screening stage. The fixed base formulation consisted of 300 g fly ash, 600 g sand, 124.5 g sodium silicate solution, 24.5 g dry NaOH, a nominal 25.6 g dispersion entry, and 10.4 g additional water, as reported in Table 4. The sodium silicate solution-to-fly ash and sand-to-fly ash mass ratios were 0.415 and 2.00, respectively. The nominal design targets were a water-to-binder ratio of 0.30 and a combined activator SiO_2_/Na_2_O molar ratio of 1.20, and these parameters were held constant across all mixtures. Total water content included the mixing water and the water contained in the sodium silicate solution, which contributed 78.435 g of water per batch, whereas the dry NaOH contributed no water. Holding the base formulation constant allowed the effects of GNP dosage and surfactant addition to be evaluated without simultaneously varying the fly ash or sand contents. For each mixture, the required GNP and surfactant masses were first dispersed in a portion of the mixing water using the same dispersion procedure applied during the preliminary screening stage. The resulting GNP–surfactant dispersion was then incorporated into the geopolymer mortar during mixing.

A total of 20 mortar mixtures were prepared, consisting of 16 GNP–surfactant mixtures, designated M1 through M16 in Table 4, and four surfactant-only control mixtures, designated C1 through C4. The two experimental variables were the nominal GNP concentration and the surfactant-to-GNP mass ratio. GNP concentrations of 0.05, 0.10, 0.50, and 1.00 wt.% relative to the water used to prepare the dispersion were evaluated. Surfactant-to-GNP mass ratios of 0.5:1, 1:1, 1.5:1, and 2:1 were also evaluated. Throughout the results and figure legends, GNP concentration (%) refers to the mass of GNP per 100 g of dispersion water and should not be interpreted as the GNP content relative to fly ash. The corresponding GNP masses were approximately 0.0043, 0.0085, 0.0426, and 0.0852 wt.% of the 300 g fly ash, respectively.

The control mixtures C1, C2, C3, and C4 contained 0.006, 0.026, 0.192, and 0.511 g, respectively, of the surfactant selected during the aqueous suspension stability screening phase. These dosages were selected to provide surfactant-only reference mixtures spanning the range of surfactant contents represented in the GNP-containing mortar matrix. Based on the listed rounded masses, the surfactant dosage in C1 corresponds to that in M1, C2 to those in M6 and M13, C3 to that in M11, and C4 to that in M16. The controls therefore provide reference points at progressively increasing surfactant dosages and allow the response of the geopolymer mortar to surfactant addition alone to be assessed across the investigated dosage range. Their purpose was to characterize the surfactant contribution at representative dosage levels rather than to provide a dosage-matched control for each individual GNP-containing mixture.

#### 2.2.4. Sample Preparation and Curing

To prepare the geopolymer mortar, Class F fly ash was first placed in a 4.7 L mechanical mixer bowl. The alkaline activator was then added, followed by the GNP–surfactant dispersion and additional water. The GNP–surfactant dispersion consisted of the prescribed GNP and surfactant masses added to water and prepared using the same mixing and ultrasonication procedure employed during the aqueous suspension screening phase. The mortar mixing sequence was adapted from ASTM C305 [32]. The constituents were mixed for 30 s at low speed, after which the full mass of sand was added gradually over the next 30 s while mixing continued at low speed. The mixer was then operated at medium speed for 30 s, followed by a 90 s rest period. During the first 15 s of the rest period, mortar adhering to the sides of the bowl was scraped down. Mixing was then resumed for an additional 60 s at medium speed.

The fresh mortar was cast into 50 mm × 50 mm × 50 mm cube molds in two layers, consistent with the specimen geometry specified in ASTM C109 [33]. Each layer was consolidated for 10 s using a vibrating table. Three replicate cubes were prepared for each mixture. The specimens were initially cured at 49 °C for 24 h and then stored at room temperature for an additional 27 days before testing at 28 days.

### 2.3. Testing Fresh and Hardened Properties of Geopolymer

#### 2.3.1. Workability

Workability was evaluated using a flow table in accordance with ASTM C1437 [34]. Fresh mortar was placed in the flow mold in two layers, with each layer tamped 20 times, and the surface was then leveled with a straightedge. The mold was lifted one minute after mixing ended, and the flow table was dropped 25 times within 15 s. The spread diameter was measured using a digital caliper. Flow was reported as the percentage increase in the average final spread diameter relative to the original base diameter of the flow mold. The resulting flow percentage was treated as an empirical measure of mortar workability and was not interpreted as an independent measure of yield stress, plastic viscosity, or thixotropy.

#### 2.3.2. Ultrasonic Pulse Velocity

Ultrasonic pulse velocity (UPV) was used as a nondestructive indicator of internal uniformity and relative changes in the geopolymer mortar matrix [35]. UPV measurements were performed in accordance with ASTM C597 [36] on 50 mm × 50 mm × 50 mm geopolymer mortar cubes. Measurements were conducted using a Pundit Ultrasonic Pulse Velocity Tester (Proceq AG, Schwerzenbach, Switzerland) equipped with 54 kHz longitudinal transducers in a direct through-transmission configuration as shown in Figure 1. Acoustic coupling gel was applied between the transducers and specimen surfaces to improve contact. The instrument had a transit-time resolution of 0.1 μs and automatic gain optimization.

Before testing, the propagation path length of each cube was measured using a digital caliper. The transducers were positioned on opposite faces of the specimen, and UPV was determined from the measured propagation path length and recorded transit time. Three replicate cubes were tested for each mixture, and the mean UPV was reported.

#### 2.3.3. Bulk Electrical Resistivity

Bulk electrical resistivity was measured to evaluate the electrical response of GNP-modified geopolymer mortar. Measurements were performed on 50 mm cube specimens using a uniaxial plate-electrode configuration adapted from ASTM C1876 [37]. The procedure was treated as an adapted bulk-resistivity method because cube specimens were used rather than the cylindrical specimens specified in the standard.

A Resipod resistivity meter (Proceq AG, Schwerzenbach, Switzerland) equipped with the bulk-resistivity attachment was used for testing. The instrument operated at 40 Hz with an applied voltage of approximately 10 V and a nominal probe spacing of 38 mm.

Measurements were performed under two moisture-conditioning states. The first was the as-cured laboratory condition at the end of the curing period described in Section 2.2.4. The specimens had been heat-cured for 24 h and then stored at room temperature for 27 days, with no additional drying treatment before testing. For the second condition, the specimens were immersed in water for 24 h, after which visible surface water was removed immediately before testing. This condition is referred to as the 24 h water-immersed, surface-dried condition. Water immersion was expected to reduce resistivity by increasing pore-liquid continuity, although pore-liquid continuity was not measured directly. The 24 h immersion period also does not establish complete pore saturation. Accordingly, comparisons between the two conditions represent responses to different moisture-conditioning states rather than a comparison between fully dry and fully saturated specimens.

The test configuration, as shown in Figure 2, consisted of two stainless-steel plate electrodes positioned on opposite specimen faces. Saturated sponge inserts were placed between the electrodes and specimen surfaces to improve electrical contact. Before testing, the sponge inserts were saturated with a calcium hydroxide solution prepared by adding 1 g of calcium hydroxide to 1 L of distilled water.

Because the sponge inserts also contributed to the measured electrical response, three instrument readings were obtained for each test condition. First, the full assembly was measured with the specimen positioned between the two sponge inserts. The resistivity of the upper and lower sponge inserts was then measured separately. The two sponge readings were subtracted from the full-assembly reading to obtain the corrected specimen response. The corrected value was then converted to specimen bulk resistivity using Equation (1).

(1) $$\rho_{\mathrm{bulk}}=\frac{\left(R_{\mathrm{assembly}}-R_{\mathrm{bottom}\mathrm{sponge}}-R_{\mathrm{top}\mathrm{sponge}}\right)}{2\pi a}\times \frac{A}{L}$$

Here, *ρ*_bulk_ denotes the specimen bulk resistivity in kΩ·cm, *R*_assembly_ is the measured resistivity of the specimen-sponge assembly (kΩ·cm), *R*_bottomsponge_ and *R*_topsponge_ denote the instrument display resistivity values in kΩ·cm for the top and bottom sponges, *a* is probe spacing, which in this case was 3.8 cm, *A* is the average cross-sectional area (cm^2^), and *L* is the specimen length (cm). Values calculated in kΩ·cm were converted to Ω·m by multiplying by 10.

#### 2.3.4. Compressive Strength

Compressive strength was measured at 28 days in accordance with ASTM C109 using 50 mm cube specimens [33]. Before testing, loose sand grains were removed from the cube faces that would contact the loading platens. The cubes were then tested using a Test Mark Industries compression testing machine with a capacity of 1112 kN. Three replicate cubes were tested for each mortar mixture, and the average compressive strength was reported.

### 2.4. Statistical Analysis

#### 2.4.1. Data Preprocessing and Standardization

Data processing was performed in R version 4.6.1 before analysis of variance (ANOVA), response surface modeling, and desirability analysis. Specimen-level observations were retained for compressive strength, UPV, and bulk electrical resistivity. Compressive strength and UPV were analyzed in their original units. For bulk electrical resistivity, measurements obtained under the as-cured condition and expressed in Ω·m were log10-transformed before statistical modeling. The transformation was used to reduce scale-dependent variance and improve the distribution and homogeneity of model residuals. Its appropriateness was assessed using residual-versus-fitted, normal Q-Q, and scale-location plots, together with the Shapiro–Wilk test for residual normality and Levene’s test for homogeneity of variance. Resistivity is therefore reported as log_10_(*ρ*_bulk_) in the statistical models, while the measured values in Ω·m are also presented to retain physical interpretability.

#### 2.4.2. Analysis of Variance

For the 16 GNP-containing mortar mixtures, two-way ANOVA was performed separately for compressive strength, ultrasonic pulse velocity UPV, and log-transformed bulk electrical resistivity to evaluate the effects of GNP concentration, surfactant-to-GNP mass ratio, and their interaction. These three terms were treated as fixed effects. Surfactant-only controls were excluded because the surfactant-to-GNP ratio is not physically defined in the absence of GNPs. When the interaction term was statistically supported, the corresponding main effects were not interpreted independently. Instead, Tukey-adjusted simple-effect comparisons were used to compare GNP concentrations within each surfactant-to-GNP ratio and surfactant-to-GNP ratios within each GNP concentration. This approach allowed the specific formulation combinations contributing to the interaction to be identified.

#### 2.4.3. Response Surface Regression

Response surface regression was used to describe fitted trends in compressive strength, ultrasonic pulse velocity, and log-transformed bulk electrical resistivity across the tested GNP concentration and surfactant-to-GNP ratio space. Before regression, both factors were centered and scaled so that the linear, interaction, and quadratic terms were estimated on comparable numerical scales. The second-order model is given in Equation (2). The fitted response surfaces were used to evaluate overall trends, curvature, and interaction patterns across the tested formulation space and to complement the categorical comparisons obtained from ANOVA. The models were used as descriptive representations of the experimental response surface and were not intended to establish physical mechanisms or to extrapolate beyond the investigated factor ranges.

(2) $$Y=\beta_{0}+\beta_{1}G_{c}+\beta_{2}R_{c}+\beta_{12}G_{c}R_{c}+\beta_{11}G_{c}^{2}+\beta_{22}R_{c}^{2}+\varepsilon$$

where Y is the response variable, $G_{c}$ is the coded GNP concentration, $R_{c}$ is the coded surfactant-to-GNP mass ratio, $\beta_{0}$ is the intercept, $\beta_{1}$ and $\beta_{2}$ are the linear coefficients, $\beta_{12}$ is the interaction coefficient, $\beta_{11}$ and $\beta_{22}$ are the quadratic coefficients, and ε is the residual error. The surfactant-only controls were not included in this ratio-based model because the surfactant-to-GNP ratio is not physically defined when GNPs are absent.

#### 2.4.4. Desirability Analysis

A desirability-function approach was used to rank the mortar mixtures by combining multiple test responses with different units and optimization directions into a single dimensionless measure. Each response was converted to an individual desirability value, $d_{i}$, ranging from 0 to 1, where 0 represents the least favorable response and 1 represents the most favorable response according to the specified criterion. The individual desirability values were then combined to obtain an overall desirability for each mixture, allowing the mixtures to be compared based on their combined performance rather than on any single test result. The approach follows the multi-response desirability framework introduced by Harrington and subsequently formalized for simultaneous response optimization by Derringer and Suich [38,39]. Additional details on the desirability-function framework are provided by Costa et al. [28].

In this study, compressive strength and UPV were treated as criteria to be maximized, while log-transformed bulk electrical resistivity was treated as a criterion to be minimized. For a criterion to be maximized, the individual desirability was calculated using the following expression:

(3) $$d_{i}=\left\{\begin{array}{cc} \begin{array}{c} 0,\\ {\left(\frac{y_{i}-L_{i}}{T_{i}-L_{i}}\right)}^{s_{i}},\\ 1, \end{array} & \begin{array}{c} y_{i}\leq L_{i}\\ L_{i}<y_{i}<T_{i}\\ y_{i}\geq T_{i} \end{array} \end{array}\right.$$

where $y_{i}$ is the measured test result, $L_{i}$ is the lower unacceptable limit, $T_{i}$ is the target value, and $s_{i}$ is a shape parameter. For a criterion to be minimized, the individual desirability was calculated as:

(4) $$d_{i}=\left\{\begin{array}{cc} \begin{array}{c} 1,\\ {\left(\frac{U_{i}-y_{i}}{U_{i}-T_{i}}\right)}^{t_{i}},\\ 0, \end{array} & \begin{array}{c} y_{i}\leq T_{i}\\ T_{i}<y_{i}<U_{i}\\ y_{i}\geq U_{i} \end{array} \end{array}\right.$$

where $T_{i}$ is the desired low value, $U_{i}$ is the upper unacceptable limit, and $t_{i}$ is a shape parameter. Equal weighting was used for all criteria. The overall desirability index was then calculated using the geometric mean of the individual desirability values:

(5) D=d1d2d313

where $d_{1}$, $d_{2}$, and $d_{3}$ represent the individual desirability values for compressive strength, UPV, and log-transformed bulk electrical resistivity, respectively. The geometric mean penalizes mixtures that perform poorly in any individual criterion; consequently, if any individual desirability value is zero, the overall desirability also becomes zero. The desirability index was used as a comparative screening metric and was not treated as direct evidence of field performance. The resulting ranking is conditional on the specified desirability limits, shape parameters, and response weights.

## 3. Results

### 3.1. GNPs Characterization Results

The commercial GNPs were characterized before mixture preparation to document their baseline morphological and structural characteristics and to verify that the supplied material exhibited features consistent with graphene nanoplatelets. Figure 3, Figure 4, Figure 5 and Figure 6 present the results of this characterization. Each technique provides complementary information on platelet morphology, particle association, graphitic structure, defect-related features, and surface chemistry.

The TEM image in Figure 3 shows a thin, translucent platelet with visible folding, wrinkling, and overlapping regions. Variations in image contrast are consistent with local differences in thickness resulting from platelet overlap or folding. The observed platelet extends across a micrometer-scale field of view, indicating a relatively large lateral dimension. These features confirm the thin, sheet-like morphology and high lateral-to-thickness aspect ratio of the GNPs. Such morphology provides a large potential contact area between platelets but can also promote folding and restacking when the particles come into close contact.

**Figure 3:**
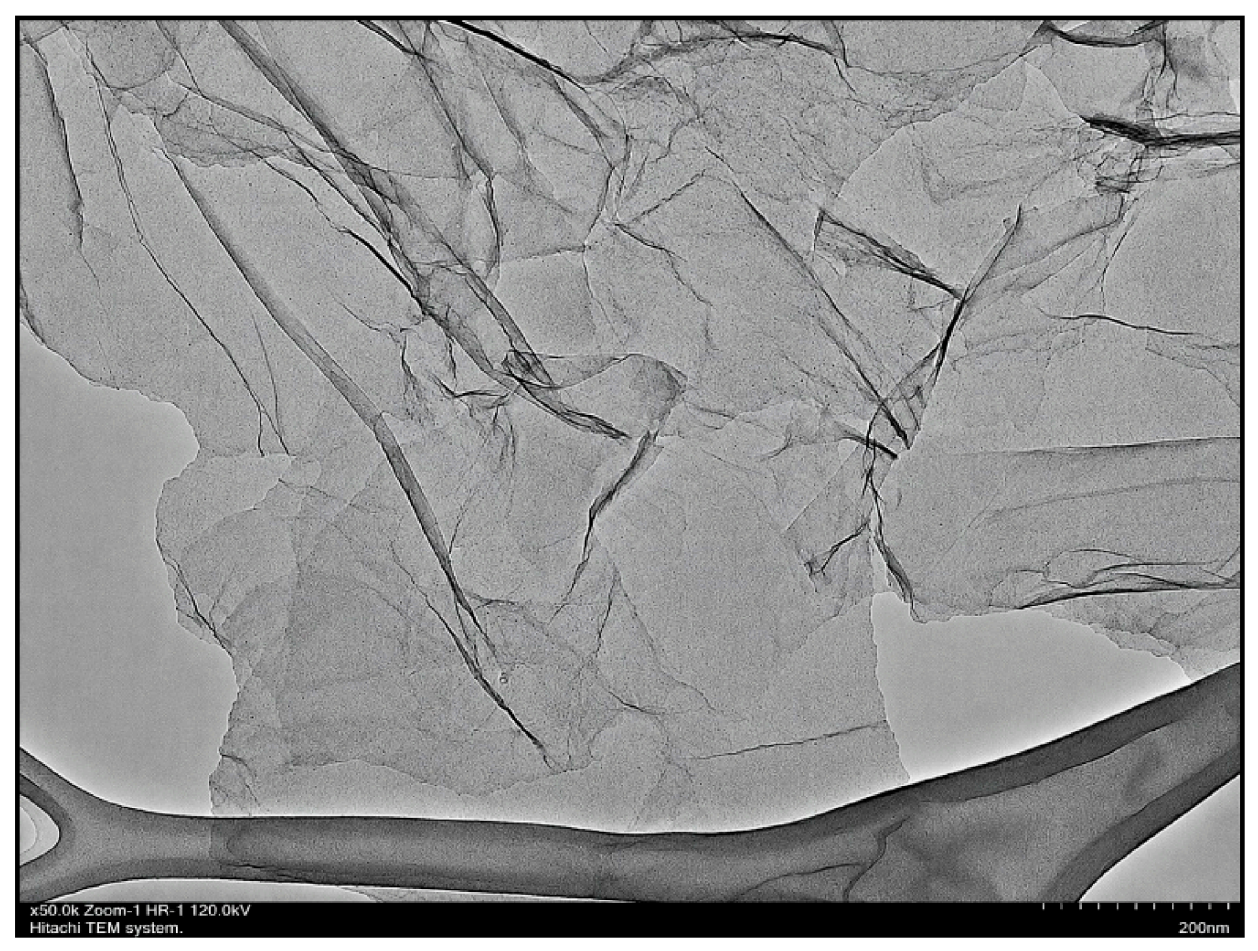
TEM image of graphene nanoplatelets.

The SEM image in Figure 4 shows plate-like features with irregular edges and micrometer-scale lateral dimensions. Several apparent features exceed 10 µm in width; however, these may represent overlapping or agglomerated platelets rather than individual GNPs. Local stacking and association among platelets are also evident in the dry powder. This platelet association indicates a tendency for the as-received GNPs to form larger assemblies, supporting the use of surfactant-assisted mixing and ultrasonication during dispersion preparation.

**Figure 4:**
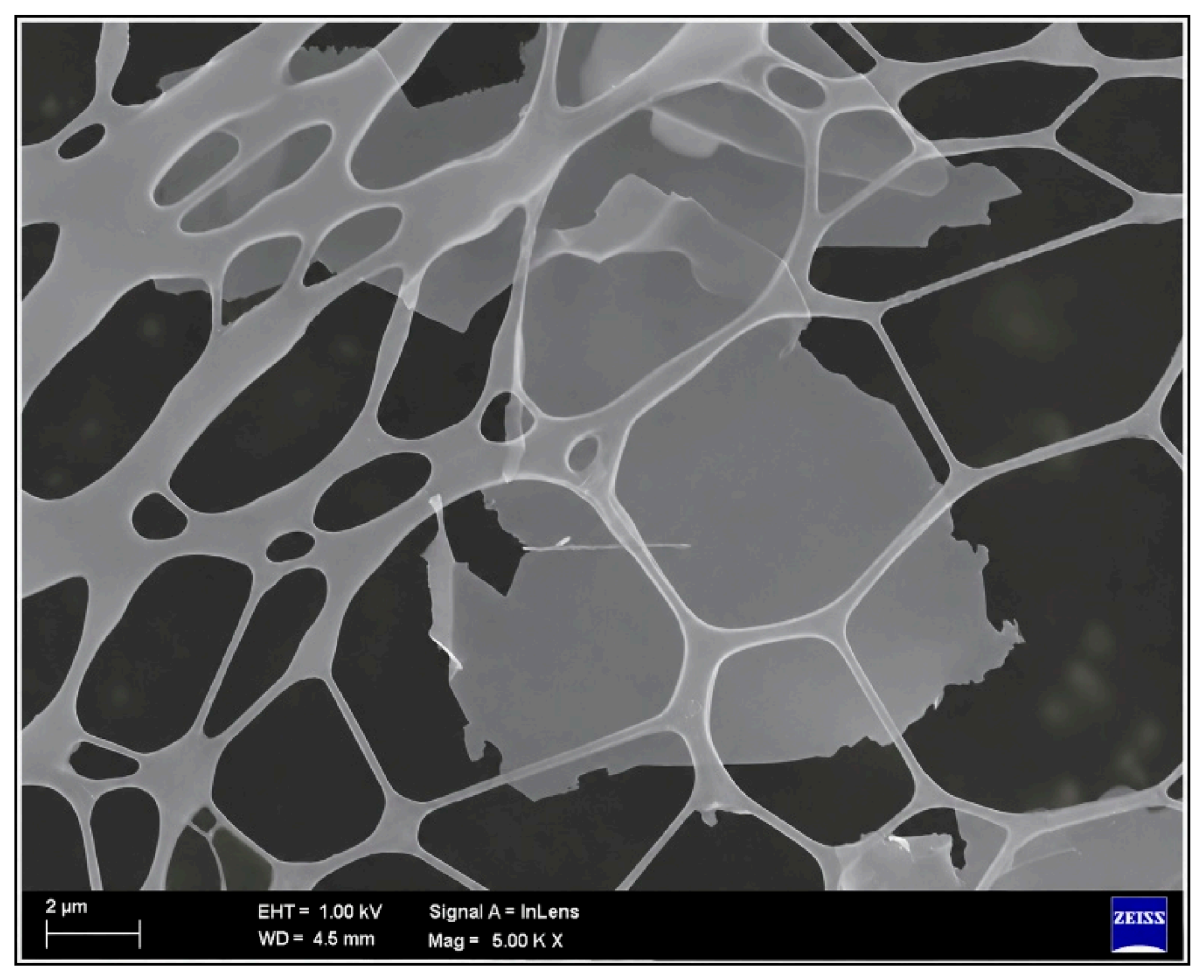
SEM image of graphene nanoplatelets.

The Raman spectrum in Figure 5 shows D, G, and 2D bands at approximately 1350, 1580, and 2720 cm⁠^−1^, respectively, consistent with predominantly graphitic sp^2^-bonded carbon. The G band is associated with in-plane vibrations of sp^2^-bonded carbon atoms, whereas the much smaller D band indicates a relatively low defect-related response. Because the D band can arise from edge defects, finite platelet dimensions, and basal-plane disorder, it should not be attributed solely to processing-induced damage. The measured *I*_2D_/*I*_G_ ratio of 0.83 is consistent with a stacked graphitic structure rather than monolayer graphene and agrees with the folded and stacked platelet morphology observed by TEM and SEM. However, Raman spectroscopy alone cannot determine the exact number of graphene layers because the 2D-band shape and intensity can also be influenced by defects, strain, doping, and measurement conditions.

**Figure 5:**
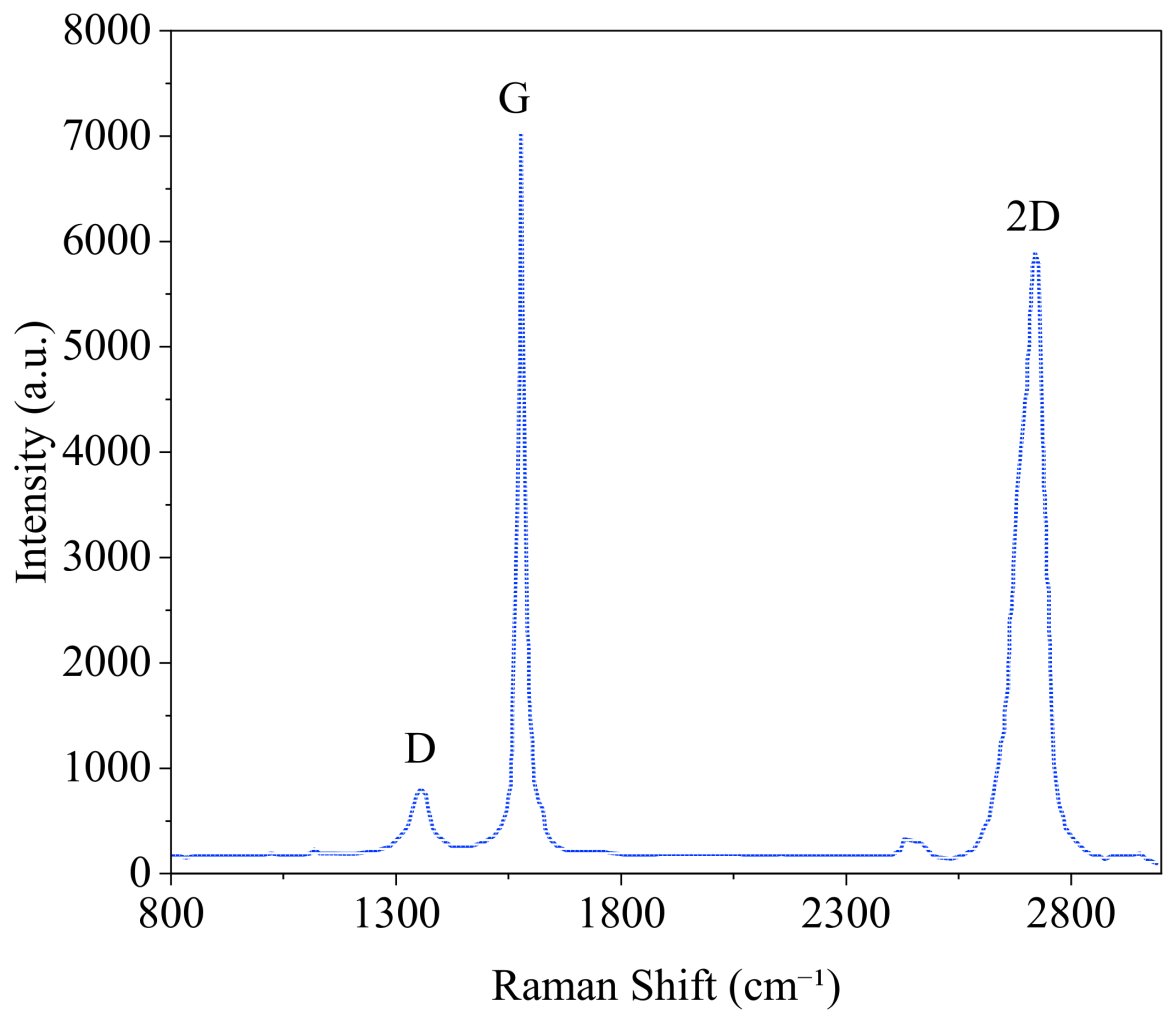
Raman spectrum of graphene nanoplatelets.

The XPS results in Figure 6 indicate that the GNP surface was predominantly carbonaceous. The survey spectrum yielded a surface composition of 92.84 at.% C and 7.16 at.% O. Deconvolution of the C 1s spectrum assigned 97.75% of the fitted carbon signal to C=C bonding and 2.25% to C–O bonding, indicating that the carbon signal was dominated by graphitic *sp*^2^-bonded carbon. Deconvolution of the O 1s spectrum assigned 14.29% of the fitted oxygen signal to C–O species and 85.71% to a component attributed to adsorbed surface H_2_O. When the oxygen contribution assigned to surface H_2_O is excluded, the normalized non-water surface composition corresponds to approximately 98.9 at.% carbon. These results are consistent with a predominantly graphitic GNP surface containing a relatively small contribution from oxygen-containing surface species. The adjusted carbon value should be interpreted as a fitting-based estimate of the non-water surface composition rather than as an independently measured bulk purity.

**Figure 6:**
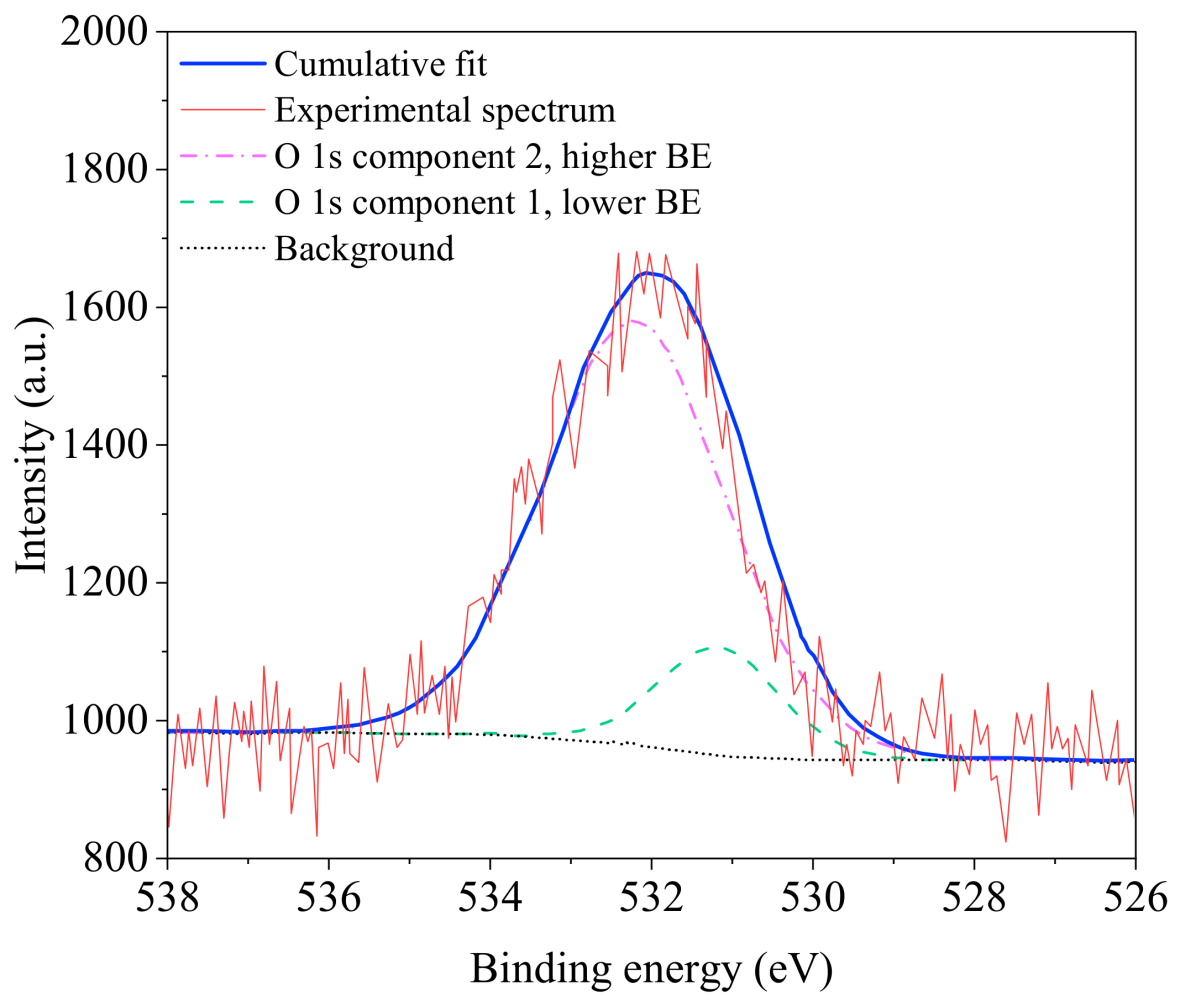
X-ray photoelectron spectroscopy (XPS) spectrum of graphene nanoplatelets.

The characterization results confirm that the commercial GNPs used in this study exhibited the expected platelet-like morphology and predominantly graphitic structure. These characteristics are directly relevant to the scope of the present study. The high-aspect-ratio platelet morphology and the observed tendency for stacking provide a physical basis for the dispersion screening conducted before mortar preparation, because platelet association can promote agglomeration in aqueous suspensions. The predominantly graphitic structure also supports the selection of GNPs as a conductive filler for evaluating changes in bulk electrical resistivity, although conductivity of the GNP powder itself was not measured. Accordingly, the characterization establishes the nature of the starting GNP material and supports the experimental design, but it does not establish the degree of GNP dispersion, interfacial bonding, or formation of conductive pathways within the hardened geopolymer mortar.

### 3.2. Evaluation of Aqueous Stability of GNP Dispersions

Phase 2 focused on the aqueous stability of GNP–surfactant dispersions before their incorporation into geopolymer mortar. The purpose of this phase was to assess whether the selected dispersion variables produced suspensions that remained sufficiently uniform for subsequent mortar preparation and to identify obvious tendencies toward settling, clustering, or phase separation. Electrical conductivity, pH, and post-storage visual observations were considered complementary screening indicators, with the visual assessment providing qualitative information on macroscopic suspension stability. These measurements were not intended to quantify nanoscale GNP dispersion or predict behavior under the highly alkaline conditions of the geopolymer system.

#### 3.2.1. Visual Screening of Aqueous Suspension Stability

Visual observation was used as a screening step in Phase 2 to assess the macroscopic stability of the aqueous GNP–surfactant dispersions before considering conductivity and pH. Figure 7, Figure 8 and Figure 9 show the post-storage appearance of the GNP dispersions prepared with SDS, TX-100, and PVP, respectively. Each formulation represents a single batch preparation; therefore, the images were used for comparative screening rather than for evaluation of batch-to-batch variability.

The SDS dispersions shown in Figure 7 generally maintained a visually continuous appearance across the tested GNP concentrations and surfactant-to-GNP ratios. The progressive darkening of the dispersions with increasing GNP concentration primarily reflects the greater GNP content and should not, by itself, be interpreted as improved stability. Importantly, the SDS formulations generally showed no widespread macroscopic particle clustering or localized deposits across the tested conditions.

**Figure 7:**
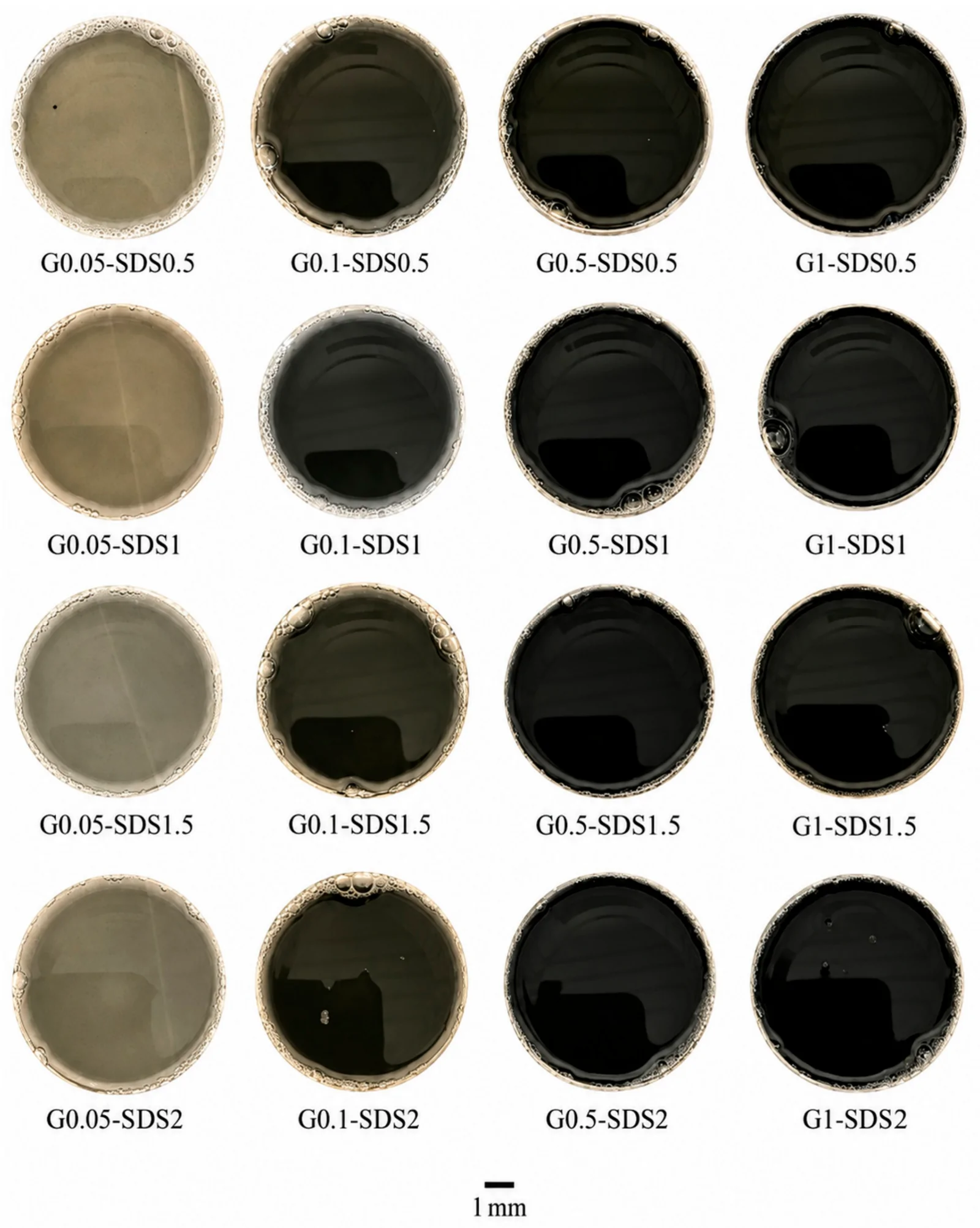
Post-storage sedimentation behavior of SDS-based GNP dispersions after 12 days of storage and transfer to beakers. Rows correspond to SDS-to-GNP ratios of 0.5, 1.0, 1.5, and 2.0. Columns correspond to GNP concentrations of 0.05%, 0.1%, 0.5%, and 1.0%.

Figure 8 shows that the TX-100 dispersions also maintained a relatively uniform macroscopic appearance across much of the screening matrix, although variations in opacity and surface appearance were observed among formulations. Based on visual observation alone, both SDS and TX-100 remained viable candidates for further consideration.

**Figure 8:**
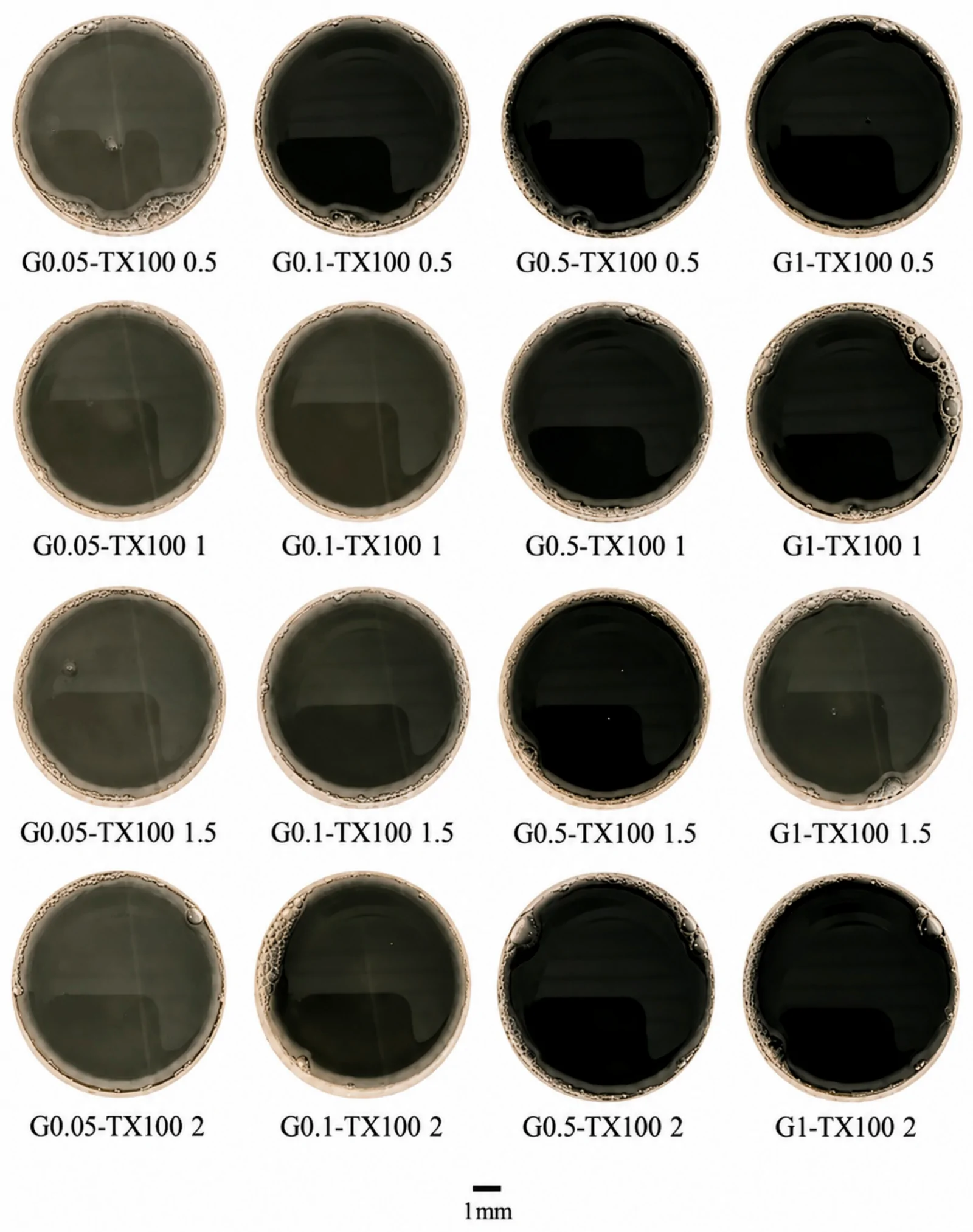
Post-storage sedimentation behavior of TX100-based GNP dispersions after 12 days of storage and transfer to beakers. Rows correspond to TX100-to-GNP ratios of 0.5, 1.0, 1.5, and 2.0. Columns correspond to GNP concentrations of 0.05%, 0.1%, 0.5%, and 1.0%.

In contrast, the PVP dispersions in Figure 9 exhibited more pronounced visible particle association and heterogeneous regions across several combinations of GNP concentration and PVP-to-GNP ratio. Distinct dark clusters and localized deposits were evident in multiple formulations, indicating less favorable macroscopic suspension behavior under the conditions evaluated. These observations provided a practical basis for excluding PVP from further mortar preparation because the objective of the screening phase was to identify a dispersing system that minimized obvious GNP association before incorporation into the geopolymer mixture.

**Figure 9:**
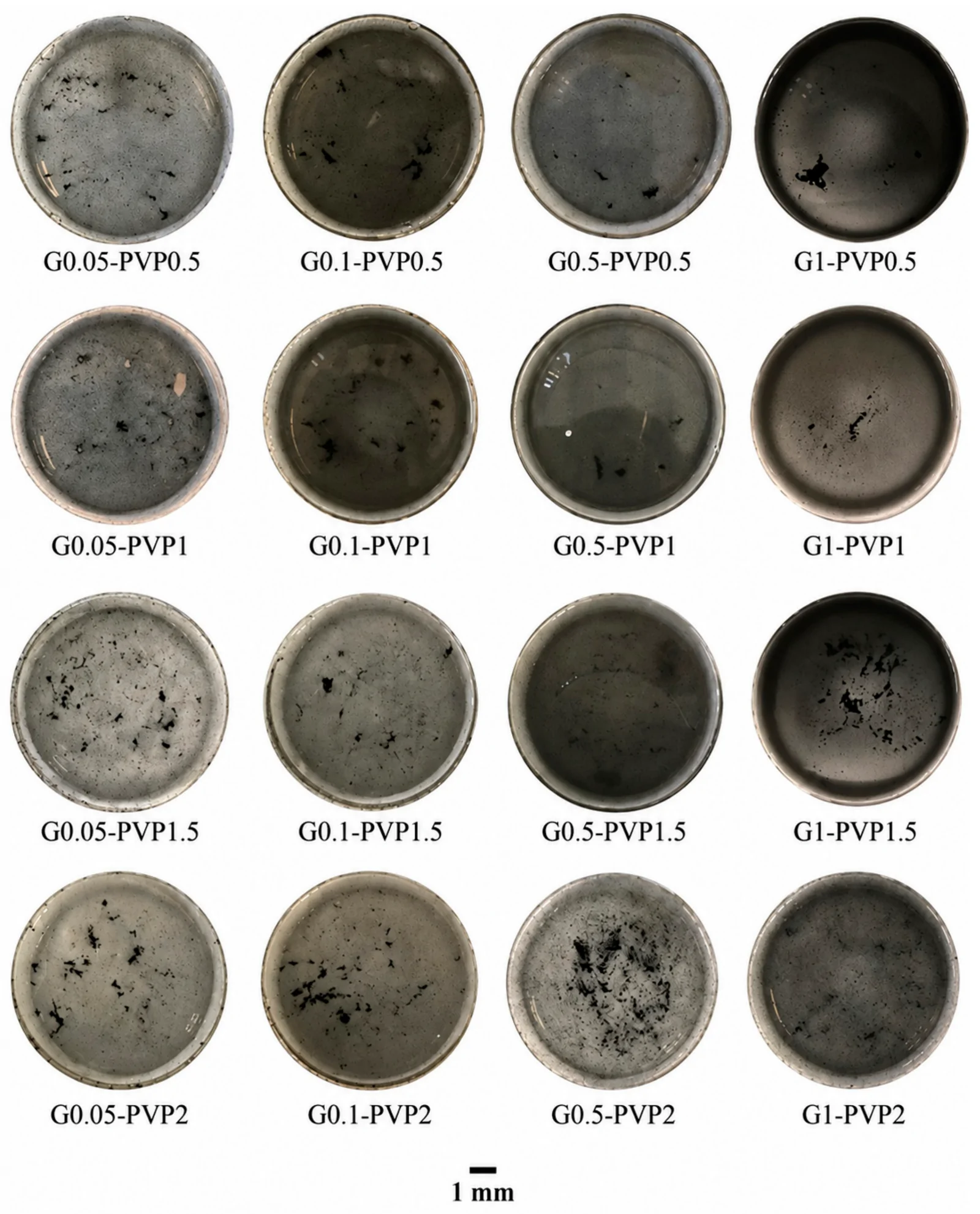
Post-storage sedimentation behavior of TX100-based GNP dispersions after 12 days of storage and transfer to beakers. Rows correspond to TX100-to-GNP ratios of 0.5, 1.0, 1.5, and 2.0. Columns correspond to GNP concentrations of 0.05%, 0.1%, 0.5%, and 1.0%.

The visual screening therefore narrowed the candidate dispersing agents from three to two, with SDS and TX-100 showing more favorable macroscopic suspension behavior than PVP. Because visual observation alone does not distinguish the physicochemical differences between the remaining SDS and TX-100 formulations, the subsequent conductivity and pH results were used as complementary screening criteria to support the final surfactant selection.

#### 3.2.2. Conductivity of Dispersions

Figure 10 presents the electrical conductivity of the aqueous GNP dispersions prepared with SDS, TX-100, and PVP at different GNP concentrations and surfactant-to-GNP mass ratios. Each formulation was prepared as a single batch; therefore, the reported values represent the measured response of the screened formulations. Following the visual screening, conductivity was used as a complementary physicochemical parameter to further compare the candidate dispersion systems.

The results in Figure 10 show that the largest conductivity differences were associated with dispersant type. SDS produced substantially higher conductivity than TX-100 and PVP across the tested conditions. For SDS, conductivity increased from approximately 115 to 300 µS/cm at 0.05% GNP, 180 to 485 µS/cm at 0.10% GNP, and 560 to 1430 µS/cm at 0.50% GNP as the surfactant-to-GNP ratio increased from 0.5 to 2.0. At 1.00% GNP, conductivity increased from approximately 860 µS/cm at a ratio of 0.5 to 1930 µS/cm at a ratio of 1.5, followed by a decrease to approximately 1660 µS/cm at a ratio of 2.0. This response shows that the electrical characteristics of the SDS formulations generally changed with formulation composition, although the increase was not proportional at the highest GNP concentration.

TX-100 and PVP exhibited much lower conductivity, generally ranging from approximately 10 to 130 µS/cm and 8 to 150 µS/cm, respectively. The higher conductivity of the SDS formulations is consistent with the ionic nature of SDS, whereas TX-100 and PVP are nonionic dispersing agents. Conductivity of aqueous SDS solutions is known to vary with surfactant concentration and can also be influenced by micellization and counterion association near the critical micelle concentration [40]. Accordingly, the conductivity response was interpreted as a characteristic of the complete aqueous formulation rather than as a direct measure of GNP dispersion quality.

At a fixed surfactant-to-GNP ratio, increasing GNP concentration also increased the absolute surfactant content. The trends in Figure 10 therefore reflect the combined composition of each formulation. Although conductive GNP networks can influence electrical behavior in solid composites as filler concentration increases [41,42,43], the aqueous conductivity measurements in this study were not intended to establish particle-to-particle contact, tunneling, or percolation among suspended GNPs. Within the screening framework, this formulation-level response was useful because the objective was to compare the actual GNP–surfactant systems subsequently considered for mortar preparation rather than to isolate the electrical contribution of each constituent. In combination with the visual observations, the conductivity results provided an additional basis for distinguishing the physicochemical behavior of the candidate dispersion systems.

#### 3.2.3. pH Values of Dispersion

Figure 11 presents the pH of the same aqueous formulations and provides a complementary measure of their acid–base environment. The results show that SDS dispersions remained mostly near neutral to mildly alkaline, ranging from approximately pH 7.0 to 8.9. TX-100 exhibited a wider range, from approximately pH 4.4 to 9.4, with higher values occurring mainly under selected low-GNP conditions and lower values at higher GNP concentrations. PVP produced the lowest pH values overall, ranging from approximately 4.1 to 7.0. At 0.50% and 1.00% GNP, SDS consistently exhibited higher pH values than TX-100 and PVP.

Within the screening design, pH was used to complement the visual and conductivity results by characterizing the chemical environment associated with each formulation. The narrower near-neutral to mildly alkaline range observed for SDS indicates a more consistent aqueous acid–base environment across the tested formulation space, whereas TX-100 showed greater variation with composition. These differences were interpreted at the formulation level because the measured pH reflects the combined influence of the dispersing agent, GNP content, and solution composition.

The strongly alkaline environment of the geopolymer activator is substantially different from that of the aqueous screening solutions. Accordingly, the pH measurements were used as a comparative screening parameter rather than as a representation of the final geopolymer chemistry. Considering the absence of visible macroscopic agglomeration, the comparatively high conductivity associated with the ionic character of SDS, and the predominantly near-neutral to mildly alkaline aqueous environment, SDS was selected as the dispersing agent for the subsequent geopolymer mortar phase. The selection was therefore based on the combined screening response rather than on any single parameter as a direct measure of GNP dispersion quality.

### 3.3. Performance Evaluation of SDS-GNP Geopolymer Mortar

#### 3.3.1. Workability Performance

Figure 12 presents the flow-table results for the SDS-GNP geopolymer mortar formulations. Workability varied with both GNP concentration and SDS dosage. At an SDS-to-GNP ratio of 0.5, flow remained near 129% for the 0.05% and 0.10% GNP mixtures, then decreased to 123.5% at 0.50% GNP and 104.4% at 1.00% GNP. Similar reductions were observed at the higher ratios. From 0.05% to 1.00% GNP, flow decreased by approximately 19% at a ratio of 0.5, 30% at a ratio of 1.0, and 24% at a ratio of 1.5. At a ratio of 2.0, the highest flow occurred at 0.10% GNP, at approximately 123.1%, followed by a decrease to 94.6% and 78.7% at 0.50% and 1.00% GNP, respectively. These trends show that higher GNP contents generally reduced workability, particularly at 0.50% and 1.00% GNP.

The SDS-only controls also showed a clear change with surfactant dosage. Flow was approximately 117.6% and 117.9% at the two lower SDS levels, then decreased to 99.0% and 73.1% at the two higher levels. This corresponds to an overall reduction of approximately 38% from the lowest to the highest SDS dosage, showing that SDS content itself contributed to the workability response.

The asterisks in Figure 12 identify GNP-containing mixtures that had the same SDS content as the corresponding SDS-only control. These matched comparisons provide a clearer indication of the change associated with GNP addition. At a ratio of 0.5, the matched 0.05% GNP mixture increased flow from 117.6% to 129.5%, corresponding to an increase of approximately 10.2%. At a ratio of 1.0, the matched 0.10% GNP mixture increased flow from 117.9% to 121.3%, an increase of about 2.9%. At a ratio of 1.5, the matched 0.50% GNP mixture produced essentially the same flow as the control, with values of 99.1% and 99.0%, respectively. At a ratio of 2.0, the matched 1.00% GNP mixture increased flow from 73.1% to 78.7%, an increase of approximately 7.7%. Thus, when SDS content was held constant, GNP addition produced either a modest increase or little change in flow rather than a consistent reduction.

The overall workability response reflects the combined influence of SDS dosage and GNP concentration. The reduction in flow at higher surfactant levels was already evident in the SDS-only controls, while increasing GNP concentration within a given formulation generally reduced flow at the higher GNP contents. At lower GNP contents, SDS-assisted particle separation and improved packing may help maintain flow, as reflected by the similar or slightly higher workability observed in the surfactant-matched comparisons. As GNP content increases, however, the high specific surface area and platelet geometry of the particles can increase liquid demand and strengthen particle–particle interactions, leading to greater resistance to flow. This interpretation is consistent with previous studies showing that the fresh behavior of graphene-modified cementitious materials depends strongly on nanoparticle dosage and dispersion condition, and that surfactant–carbon nanomaterial systems can modify interparticle interactions rather than simply increase flow [20,44,45].

#### 3.3.2. Compressive Strength Developement

Figure 13 presents the 28-day compressive strength of the SDS-GNP geopolymer mortar mixtures. Mean strength ranged from approximately 5.5 to 16.8 MPa and varied with both GNP concentration and SDS-to-GNP ratio. The highest mean strength was approximately 16.8 MPa at 0.10% GNP and an SDS-to-GNP ratio of 1.5. A second high value of approximately 15.6 MPa was obtained at 0.05% GNP and a ratio of 2.0. In contrast, the 1.00% GNP formulations generally exhibited lower strength across the tested ratios. The results therefore indicate that increasing GNP concentration did not produce a continuous improvement in compressive strength.

The SDS-only controls show that their strength decreased from approximately 14.6 MPa at the lowest SDS content to 12.8, 9.5, and 7.0 MPa as SDS dosage increased. This represents an overall reduction of about 52%, indicating that increasing SDS content alone was associated with a progressive reduction in compressive strength. The contribution associated with GNP can then be examined by comparing GNP-containing mixtures with controls having the same SDS content and no GNP. At the lowest matched condition, adding 0.05% GNP reduced strength by about 28%, from 14.6 to 10.5 MPa. At the next two matched conditions, 0.10% and 0.50% GNP increased strength by approximately 13%, from 12.8 to 14.4 MPa and from 9.4 to 10.6 MPa, respectively. At the highest matched condition, 1.00% GNP reduced strength by about 21%, from 7.0 to 5.5 MPa. These comparisons show that SDS and GNP affected strength differently. Increasing SDS dosage was associated with a general downward trend in the control mixtures, while the response associated with GNP depended on dosage. Moderate GNP contents produced modest strength gains relative to their SDS-matched controls, whereas the lowest and highest matched GNP contents did not improve strength.

The 95% confidence intervals in Figure 13 show that specimen-level variability also differed among formulations. Most mixtures exhibited relatively narrow intervals, while selected low-GNP formulations showed wider intervals. Because each mean was based on three replicate cubes, the confidence intervals are sensitive to moderate differences among individual specimens. Local variation in GNP distribution, particle association, entrapped air, and consolidation may contribute to this variability. The mean strength values were therefore interpreted together with their associated uncertainty.

The overall response is consistent with a balance between beneficial particle effects at moderate GNP contents and increasing particle interaction at higher contents. At suitable dosages, improved particle separation may promote packing, nucleation, and local crack-bridging effects. As GNP content increases, greater platelet association may introduce local discontinuities and reduce effective load transfer. The influence of SDS on fresh-state behavior and subsequent matrix formation also contributes to the hardened response. These trends are consistent with previous studies showing that the mechanical contribution of graphene-family additives depends strongly on dosage and dispersion condition [16,20,46], while surfactant–carbon nanomaterial systems can alter fresh behavior in ways that affect hardened properties [45]. The lower strengths observed at higher GNP concentrations also coincided with the lower flow values in Figure 12 and the generally lower UPV values in Figure 14, indicating a consistent formulation-level response across the fresh and hardened measurements.

Published graphene-modified geopolymer systems show a wide range of strength responses. Abiodun et al. [24] reported a 28-day compressive strength of 63.5 MPa at 0.07 wt.% pristine graphene relative to the precursor. Ranjbar et al. [16] reported a 1.44-fold increase in compressive strength at 1 wt.% GNP, while Sajjad et al. [7] reported an approximately 10% increase at 1% graphene in a slag/fly ash mortar. These values provide useful context but are not direct benchmarks because graphene type, dosage basis, precursor chemistry, activator composition, curing regime, and reference formulations differ among studies. Within the scope of the present work, the measured strength range of 5.5 to 16.8 MPa establishes the mechanical response of the selected SDS-GNP geopolymer formulations and provides the mechanical context for the subsequent evaluation of their electrical behavior.

#### 3.3.3. Ultrasonic Pulse Velocity Response

The 28-day UPV results in Figure 14 complement the compressive-strength results by providing a comparative indicator of internal continuity and stiffness across the SDS-GNP geopolymer mortar formulations. Under the same testing conditions, higher UPV was interpreted as indicating greater internal continuity and stiffness, whereas lower UPV was associated with greater internal heterogeneity or discontinuity. The response was formulation-dependent and broadly consistent with the strength trends. Lower GNP concentrations, particularly 0.05% and 0.10%, generally produced the highest UPV values. The maximum value, approximately 3.0 km/s, occurred at 0.05% GNP and an SDS-to-GNP ratio of 1.5. A comparable value of approximately 2.95 km/s was obtained at 0.10% GNP and a ratio of 0.5. In contrast, the 1.00% GNP formulations ranged from approximately 2.08 to 2.50 km/s and were among the lowest across most SDS-to-GNP ratios.

Comparing the SDS-only controls with their matched GNP-containing counterparts shows that GNP addition does not produce a uniform effect on internal continuity. The SDS-only controls isolate the influence of surfactant dosage from the contribution of GNP. For the two lower SDS levels, the UPV remained stable between 2.6 and 2.7 km/s before dropping to approximately 2.35 and 2.2 km/s at higher dosages. Overall, the control UPV decreased by roughly 17% from the lowest to the highest SDS level. Although the response was not strictly monotonic, this downward trend suggests that higher SDS concentrations reduce internal continuity at the upper end of the tested dosage range. At the lowest matched condition, adding 0.05% GNP increased the UPV by about 6% from approximately 2.6 to 2.8 km/s. The 0.10% GNP addition produced essentially no change, keeping both values near 2.7 km/s. The third matched condition of 0.50% GNP yielded another 6% increase, raising the UPV from 2.35 to 2.50 km/s. Conversely, the highest concentration of 1.00% GNP reduced the UPV by about 5% from 2.2 to 2.1 km/s. These findings confirm that low and intermediate GNP contents provide modest increases relative to their SDS-matched controls, whereas the highest GNP content directly reduces the UPV.

The 95% confidence intervals indicate that specimen-level variability differed among formulations, although most intervals were relatively narrow. Because each mean was based on three replicate cubes, the intervals are sensitive to differences among individual specimens and were considered when interpreting the mean values. The generally higher UPV at lower GNP concentrations is consistent with greater internal continuity, while the lower values at higher GNP contents suggest greater internal heterogeneity. Differences in particle packing, pore structure, entrapped air, interfacial contact, and matrix stiffness may contribute to this behavior. Similar dosage- and dispersion-dependent effects of graphene-family nanomaterials have been reported in cementitious and geopolymeric systems [20,44,46]. At suitable dosages, improved particle distribution may contribute to a more continuous matrix, whereas higher particle contents can increase platelet association and local heterogeneity. The UPV results therefore provide complementary evidence that the internal condition of the geopolymer mortar depended on the GNP-SDS formulation and that increasing GNP content beyond an appropriate range did not provide further improvement.

#### 3.3.4. Bulk Electrical Resistivity

Electrical resistivity was evaluated under two moisture-conditioning states to determine how the GNP-SDS formulations responded under different levels of pore-liquid availability. Figure 15 presents the as-cured results, while Figure 16 presents the results after 24 h water immersion followed by surface drying. The as-cured specimens generally exhibited resistivity values of approximately 60 to 180 Ω·m, whereas the 24 h water-immersed specimens were substantially lower, generally ranging from approximately 5 to 29 Ω·m. This reduction after water immersion is consistent with greater ionic conduction through the pore solution and shows that moisture condition had a strong influence on the measured electrical response.

The as-cured results in Figure 15 show a nonmonotonic response to GNP concentration. The highest mean resistivity, approximately 180 Ω·m, occurred at 0.50% GNP and an SDS-to-GNP ratio of 0.5. Other relatively high values occurred at 0.05% and 0.10% GNP across several ratio groups. The surfactant-matched comparisons provide a clearer indication of the response associated with GNP addition. At a ratio of 0.5, adding 0.05% GNP increased resistivity from approximately 66 to 74 Ω·m. At a ratio of 1.0, the matched 0.10% GNP formulation increased resistivity from approximately 72 to 110 Ω·m. At a ratio of 1.5, the matched 0.50% GNP formulation increased resistivity from approximately 66 to 91 Ω·m. At a ratio of 2.0, the matched 1.00% GNP formulation increased resistivity from approximately 59 to 84 Ω·m. Thus, GNP addition did not reduce as-cured resistivity within the matched formulations. The nonmonotonic response instead suggests that bulk resistivity was governed by competing contributions from pore-liquid conduction, GNP distribution and contact, platelet association, pore structure, and electrode contact rather than by a single continuous GNP pathway.

The 24 h water-immersed results in Figure 16 show a different absolute resistivity range but a similarly formulation-dependent response. Resistivity of the SDS-only controls increased across the control series from approximately 5 Ω·m at the lowest SDS level to approximately 26 Ω·m at the highest. Within the surfactant-matched comparisons, the 0.05% GNP formulation at a ratio of 0.5 increased resistivity from approximately 5 to 11 Ω·m, while the matched 0.10% GNP formulation at a ratio of 1.0 increased it from approximately 9 to 29 Ω·m. At a ratio of 1.5, the matched 0.50% GNP formulation increased resistivity from approximately 18 to 26 Ω·m. At a ratio of 2.0, the matched 1.00% GNP formulation produced a resistivity close to that of its control, at approximately 26 Ω·m. The lower overall values after immersion are consistent with increased pore-liquid continuity, while the variation among formulations indicates that moisture condition and mixture composition acted together to control the measured response. The 24 h immersion condition was used to provide a consistent moisture-conditioning state and was not intended to establish complete saturation.

The results show that a consistent reduction in resistivity was not observed with increasing GNP concentration under either moisture condition. This is important because a monotonic reduction might be expected if bulk electrical transport were controlled primarily by a continuous GNP network. The present results instead indicate that ionic transport through the pore solution remained an important contributor and that GNP incorporation may simultaneously influence particle connectivity, pore structure, and ionic transport pathways. Under the as-cured condition, residual pore liquid remains part of the measured electrical response, while the immersed condition increases the contribution of pore-liquid conduction. The two conditioning states therefore provide complementary information on how the formulations respond under different moisture conditions rather than isolating purely electronic conduction through the GNPs.

The comparatively wide 95% confidence intervals observed for selected formulations also indicate greater specimen-level variability in resistivity than in UPV. This behavior is consistent with the sensitivity of electrical measurements to local moisture distribution, pore connectivity, GNP distribution, and conductive-path continuity [11,47]. Within the scope of this study, the resistivity measurements establish that the GNP-SDS geopolymer mortars exhibit formulation- and moisture-dependent electrical behavior. They do not, however, show that increasing GNP concentration alone consistently improves electrical performance. Rather, the results provide the electrical basis for identifying promising formulations for subsequent application-specific evaluation, such as sensing, Joule heating, or electromagnetic response, where those functions would need to be measured directly.

### 3.4. Statistical Evaluation of Experimental Results

#### 3.4.1. Factor Effects and Interaction Structure

Two-way ANOVA was used to evaluate the effects of GNP concentration, SDS-to-GNP ratio, and their interaction on compressive strength, UPV, and log-transformed bulk electrical resistivity. The surfactant-only controls were excluded because the SDS-to-GNP ratio is not physically defined when GNP is absent.

A statistically significant GNP concentration × SDS-to-GNP ratio interaction was observed for all three responses (p<0.001). Therefore, the effects of GNP concentration and SDS-to-GNP ratio were interpreted in combination rather than as independent effects. Tukey-adjusted simple-effect comparisons were subsequently used to identify differences among GNP concentrations within each SDS-to-GNP ratio.

For compressive strength, GNP concentration had the largest partial effect size among the fitted model terms, F(3|32)=55.604, p<0.001, $\eta_{p}^{2}=0.839$. The interaction was also strongly supported, F(9|32)=7.175, p<0.001, $\eta_{p}^{2}=0.669$, while the SDS-to-GNP ratio had a smaller partial effect size, F(3|32)=4.208, p=0.013, $\eta_{p}^{2}=0.283$. Tukey comparisons showed that at a ratio of 0.5, the 1.00% GNP mixture had lower strength than the 0.05% and 0.10% mixtures. At a ratio of 1.0, the 0.10% mixture had higher strength than the 0.50% and 1.00% mixtures, while the 1.00% mixture was also lower than the 0.05% and 0.50% mixtures. At a ratio of 1.5, the 0.10% mixture had higher strength than the 0.05%, 0.50%, and 1.00% mixtures (p<0.001 for all three comparisons). At a ratio of 2.0, strength decreased from the 0.05% mixture to the higher GNP levels, with the 0.05% mixture exceeding the 0.10%, 0.50%, and 1.00% mixtures. These results confirm that the influence of GNP concentration depended strongly on the SDS-to-GNP ratio.

For UPV, GNP concentration again had the largest partial effect size, F(3|32)=94.228, p<0.001, $\eta_{p}^{2}=0.898$. SDS-to-GNP ratio, F(3|32)=13.766, p<0.001, $\eta_{p}^{2}=0.563$, and the interaction, F(9|32)=6.638, p<0.001, $\eta_{p}^{2}=0.651$, were also statistically significant. Tukey comparisons generally showed higher UPV at the lower GNP concentrations. At a ratio of 0.5, both 0.05% and 0.10% GNP produced higher UPV than 0.50% and 1.00% GNP. At a ratio of 1.0, both 0.05% and 0.10% GNP produced higher UPV than 1.00% GNP. At a ratio of 1.5, the 0.05% mixture exceeded the 0.10%, 0.50%, and 1.00% mixtures, while the 0.10% mixture also exceeded the 0.50% and 1.00% mixtures. A similar pattern occurred at a ratio of 2.0, where 0.05% GNP exceeded all higher concentrations and 0.10% GNP exceeded both 0.50% and 1.00% GNP. These results support the observed tendency toward higher UPV at lower GNP contents, although UPV alone cannot distinguish among effects associated with GNP distribution, entrapped air, and matrix heterogeneity.

For log-transformed bulk electrical resistivity, GNP concentration, F(3|32)=18.004, p<0.001, $\eta_{p}^{2}=0.628$, SDS-to-GNP ratio, F(3|32)=11.850, p<0.001, $\eta_{p}^{2}=0.526$, and their interaction, F(9|32)=5.087, p<0.001, $\eta_{p}^{2}=0.589$, were all statistically significant. The Tukey results showed a less systematic pattern than for strength and UPV. At a ratio of 0.5, the 0.10% mixture had lower log-transformed resistivity than the 0.50% and 1.00% mixtures, while the 0.05% mixture was also lower than the 1.00% mixture. At a ratio of 1.0, the 0.05% mixture had lower resistivity than the 0.10%, 0.50%, and 1.00% mixtures. The same pattern occurred at a ratio of 1.5, where the 0.05% mixture was lower than all three higher GNP concentrations. At a ratio of 2.0, only the difference between 0.10% and 1.00% GNP was statistically significant. The absence of a consistent concentration-dependent decrease across the four ratios agrees with the resistivity trends discussed previously and indicates that the electrical response depended on the specific GNP-SDS formulation rather than on GNP concentration alone.

The statistical results confirm that the performance of the geopolymer mortar was governed by the specific combination of GNP concentration and SDS-to-GNP ratio rather than by either factor alone. Moderate GNP contents generally provided the most favorable mechanical and ultrasonic responses, whereas the electrical response remained formulation-dependent and did not show a consistent concentration-driven improvement.

#### 3.4.2. Response Surface Interpretation

Response surface regression was used to summarize the overall variation in compressive strength, UPV, and log-transformed bulk resistivities across the tested GNP concentration and SDS-to-GNP ratio ranges. The fitted models were intended to describe broad direction, curvature, and combined factor behavior rather than reproduce every difference among the sixteen individual formulations. The estimated coefficients, *R*^2^, and root mean square error (RMSE) are reported in Table 5.

For compressive strength, the linear coded-GNP coefficient was negative ($b_{1}=-2.624$, p<0.001), indicating an overall decrease in fitted strength as GNP concentration increased. The linear SDS-to-GNP ratio term was not statistically significant. The ratio-squared and GNP concentration × SDS-to-GNP ratio terms had 0.05≤p<0.10 and therefore were not statistically significant at α=0.05. The model produced an $R^{2}$ of 0.622 and an RMSE of 1.996 MPa, indicating that the quadratic surface captured the general response but left substantial formulation-level variation unexplained.

As shown in Figure 17, the fitted surface places the highest compressive strengths in the lower-GNP region and shows a general decrease in strength toward 1.00% GNP. The surface also indicates some dependence on the SDS-to-GNP ratio, particularly at the lower GNP concentrations, although this effect is less pronounced than the overall GNP concentration trend. This agrees with the experimental results, where selected low and intermediate GNP formulations produced higher strengths, while the 1.00% GNP mixtures generally exhibited lower values.

The response-surface model should be interpreted together with the factorial ANOVA. The categorical ANOVA identified a statistically significant GNP concentration × SDS-to-GNP ratio interaction, whereas the single bilinear interaction term in the quadratic regression was not significant at α=0.05. These findings are not contradictory because the two models represent interaction differently. The factorial ANOVA allows the effect of GNP concentration to vary separately at each tested SDS-to-GNP ratio, while the quadratic model approximates the sixteen formulations using a smooth continuous surface. The response surface therefore provides a useful visualization of the overall trend, while the ANOVA and Tukey comparisons provide stronger evidence of the formulation-specific differences.

For UPV, the response surface model showed a stronger fit than the compressive-strength model. The linear coefficients for GNP concentration and SDS-to-GNP ratio were both negative and statistically significant, with $b_{1}=-0.259$ (p<0.001) and $b_{2}=-0.063$ (p<0.01), respectively. The interaction term was also negative and statistically significant, with $b_{12}=-0.063$ (p<0.01), indicating that the effect of GNP concentration depended on the SDS-to-GNP ratio. The positive quadratic GNP term, $b_{11}=0.082$ (p<0.01), indicates curvature along the GNP concentration axis. Overall, the fitted response was characterized by lower UPV toward higher GNP concentrations, particularly when combined with higher SDS-to-GNP ratios. The model had an $R^{2}$ of 0.791 and an RMSE of 0.120 km/s, providing the strongest fit among the three response-surface models.

The UPV response surface in Figure 18 places the highest fitted values in the low-GNP region, particularly at intermediate SDS-to-GNP ratios, and lower values toward the high-GNP and high-ratio region. This broad trend agrees with the factorial ANOVA and Tukey comparisons, which showed that lower GNP concentrations generally produced higher UPV than the 0.50% and 1.00% formulations. The response is consistent with differences in internal continuity among the mixtures. Higher GNP contents may promote platelet association, entrapped air, or other matrix discontinuities that reduce ultrasonic wave transmission. However, UPV alone cannot distinguish among effects associated with GNP distribution, air content, pore structure, matrix stiffness, moisture condition, or transducer contact.

For log-transformed resistivity, the linear coefficients for GNP concentration and SDS-to-GNP ratio were positive and statistically significant, with $b_{1}=0.125$ (p<0.001) and $b_{2}=0.059$ (p<0.01), respectively. However, these coefficients represent the fitted slopes at the coded origin and should not be interpreted as evidence of monotonic increases across the full experimental range. The negative SDS-to-GNP quadratic term was statistically significant ($b_{22}=-0.067$, p<0.05), while the GNP quadratic term was not statistically significant. The interaction term showed weak statistical support ($b_{12}=-0.033$, 0.05≤p<0.10). Examination of the fitted surface showed that log-resistivity generally increased with GNP concentration over much of the tested range, but the response flattened and, at higher SDS-to-GNP ratios, decreased near the upper GNP range. The model had an $R^{2}$ of 0.528 and an RMSE of 0.125, representing the lowest explanatory capacity among the three response-surface models.

Figure 19 summarizes the fitted log-resistivity response within the tested design space. The broad increase toward higher GNP concentrations is therefore not uniform across all SDS-to-GNP ratios. This behavior agrees with the factorial ANOVA and Tukey comparisons, which showed that resistivity depended on the specific combination of GNP concentration and SDS-to-GNP ratio rather than on GNP concentration alone. The fitted response does not support a simple assumption that increasing GNP necessarily decreases bulk resistivity. It also cannot distinguish changes in GNP particle connectivity from changes in pore-liquid conduction or pore structure and does not establish the presence or absence of an electrically percolated GNP network.

#### 3.4.3. Desirability-Based Mixture Screening

Figure 20 presents the desirability ranking of the GNP-containing mixtures based on compressive strength, UPV, and log-transformed bulk electrical resistivity. Compressive strength and UPV were assigned maximize objectives, while log-transformed resistivity was assigned a minimize objective. Each response was linearly scaled between the minimum and maximum values observed across the complete set of tested formulations, including the surfactant-only controls. The three individual desirability values were then combined using an equally weighted geometric mean. Lower resistivity was selected as a screening preference for subsequent electrical-response evaluation and does not by itself indicate improved sensing, heating, or electromagnetic-shielding performance.

Among the GNP-containing mixtures shown inFigure 20, M2 had the highest overall desirability, approximately 0.73. M2 contained 0.10% GNP relative to dispersion water and an SDS-to-GNP ratio of 0.5. The next highest-ranked mixtures were M1 and M9, both containing 0.05% GNP, followed by M5, M14, and M13. The ranking therefore generally favored the lower GNP concentrations, while no single SDS-to-GNP ratio was consistently preferred across the highest-ranked mixtures.

The desirability values should be interpreted as a comparative screening metric rather than as evidence of a unique optimum. The ranking depends on the selected responses, the specified maximize or minimize directions, the observed scaling limits, and the equal weighting assigned to the three criteria. A different application objective or weighting scheme could therefore produce a different ranking. In addition, the ranking compares the measured performance of complete formulations and does not isolate the individual contributions of GNP and SDS.

The zero overall desirability values for M6 and M16 do not indicate zero measured performance. M6 reached the least favorable end of the selected resistivity scale, while M16 reached the least favorable end of the strength and UPV scales. Because the overall desirability was calculated as the geometric mean of the three individual desirabilities, any individual desirability of zero produces an overall value of zero. This approach prevents a strong result in one response from fully compensating for a poor result in another.

The desirability ranking should be treated as a screening result, not as proof of a mechanistic optimum. It depends on the selected responses, target directions, scaling limits, and equal weighting. A different application target could change the ranking. For example, a mixture selected primarily for mechanical performance could rank differently from a mixture selected primarily for lower resistivity. Therefore, the ranking is most defensible as a decision-support tool for selecting candidate formulations for follow-up testing.

Within the tested factor range and selected desirability criteria, the screening results favor selected mixtures containing 0.05–0.10% GNP relative to dispersion water, corresponding to approximately 0.0042–0.0085 wt.% relative to fly ash. M2 was the highest-ranked GNP-containing formulation under the selected criteria. However, this ranking should not be interpreted as a universal material optimum because it is conditional on the selected performance objectives and does not include uncertainty in the desirability calculation or independent batch validation.

## 4. Conclusions

Graphene nanoplatelets offer a potential means of modifying the mechanical and electrical behavior of geopolymer composites, but their performance depends on dosage, dispersion, and surfactant interactions. This study characterized commercial GNPs, screened SDS, TX-100, and PVP as dispersing agents, evaluated the effects of GNP concentration and SDS-to-GNP ratio on geopolymer mortar properties, and identified promising formulations using statistical and multi-response analyses.

Screening 48 aqueous GNP–surfactant formulations supported the selection of SDS for the mortar phase based on macroscopic observations, conductivity, and pH. SDS showed favorable visible stability, a strong ionic conductivity response, and a relatively consistent near-neutral to mildly alkaline aqueous environment. These measurements supported formulation selection but did not quantify nanoscale dispersion or predict behavior in the alkaline geopolymer matrix.

Mortar performance depended on the combined GNP concentration and the SDS-to-GNP ratio. Increasing the GNP concentration did not produce monotonic improvements in flow, compressive strength, UPV, or resistivity. Furthermore, comparing the SDS-only controls with their surfactant-matched counterparts showed that the response to GNP addition depended heavily on the accompanying SDS dosage.

The geopolymer mortar exhibited compressive strengths ranging from approximately 5.5 to 16.8 MPa. Performance did not improve monotonically with increasing GNP concentration. Lower and intermediate GNP concentrations generally produced higher strength than the 1.00% formulations. UPV showed a similar pattern, with the 0.05% and 0.10% GNP mixtures generally producing higher values than mixtures containing 0.50% and 1.00% GNP. Statistical analysis confirmed significant interactions between GNP concentration and SDS-to-GNP ratio for strength, UPV, and resistivity, indicating that performance depended on the specific combination of GNP and surfactant rather than on GNP concentration alone.

Moisture condition had a strong influence on electrical resistivity. After 24 h water immersion followed by surface drying, resistivity was substantially lower than under the as-cured condition, consistent with increased pore-liquid conduction. Increasing GNP concentration did not consistently reduce resistivity. Response-surface analysis further showed a formulation-dependent and nonmonotonic electrical response, suggesting that the measured resistivity reflected combined effects of GNP distribution, particle contact, pore structure, and ionic transport.

The desirability analysis combined compressive strength, UPV, and log-transformed resistivity for the 16 GNP-containing mixtures, with strength and UPV maximized and resistivity minimized. M2 had the highest overall desirability and contained 0.10% GNP relative to dispersion water with an SDS-to-GNP ratio of 0.5. Most of the higher-ranked formulations contained 0.05–0.10% GNP, corresponding to approximately 0.0042–0.0085 wt.% relative to fly ash. These results identify a promising low-GNP formulation range for further evaluation rather than a universal optimum.

Further work should link these formulation-level responses to the hardened matrix through SEM/EDS, XRD, TGA, calorimetry, and pore-structure characterization. Independent batch replication and direct testing of sensing, resistive heating, and electromagnetic responses are also needed to evaluate application-specific performance.

## Figures and Tables

**Figure 1:**
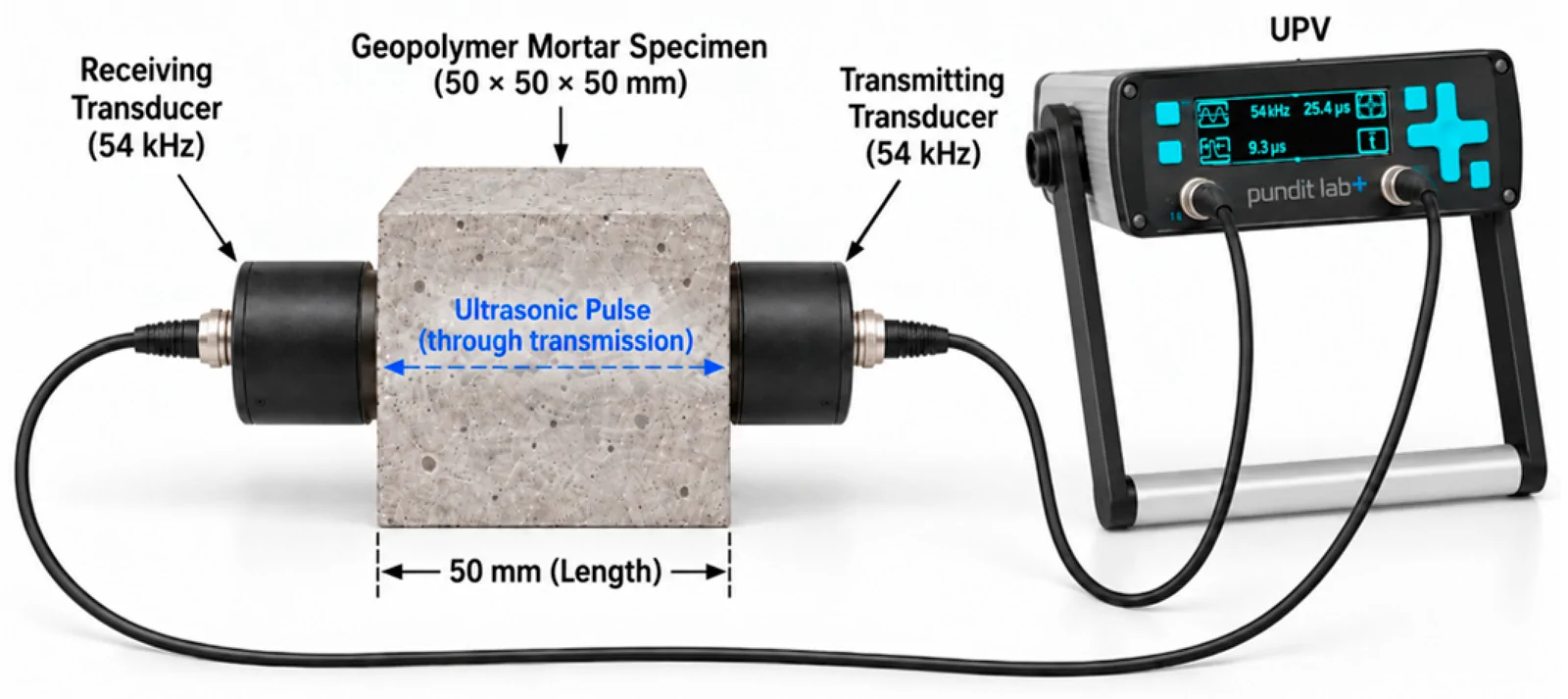
Ultrasonic pulse velocity test setup for 50 mm geopolymer mortar cubes using 54 kHz transmitting and receiving transducers in a direct through-transmission configuration.

**Figure 2:**
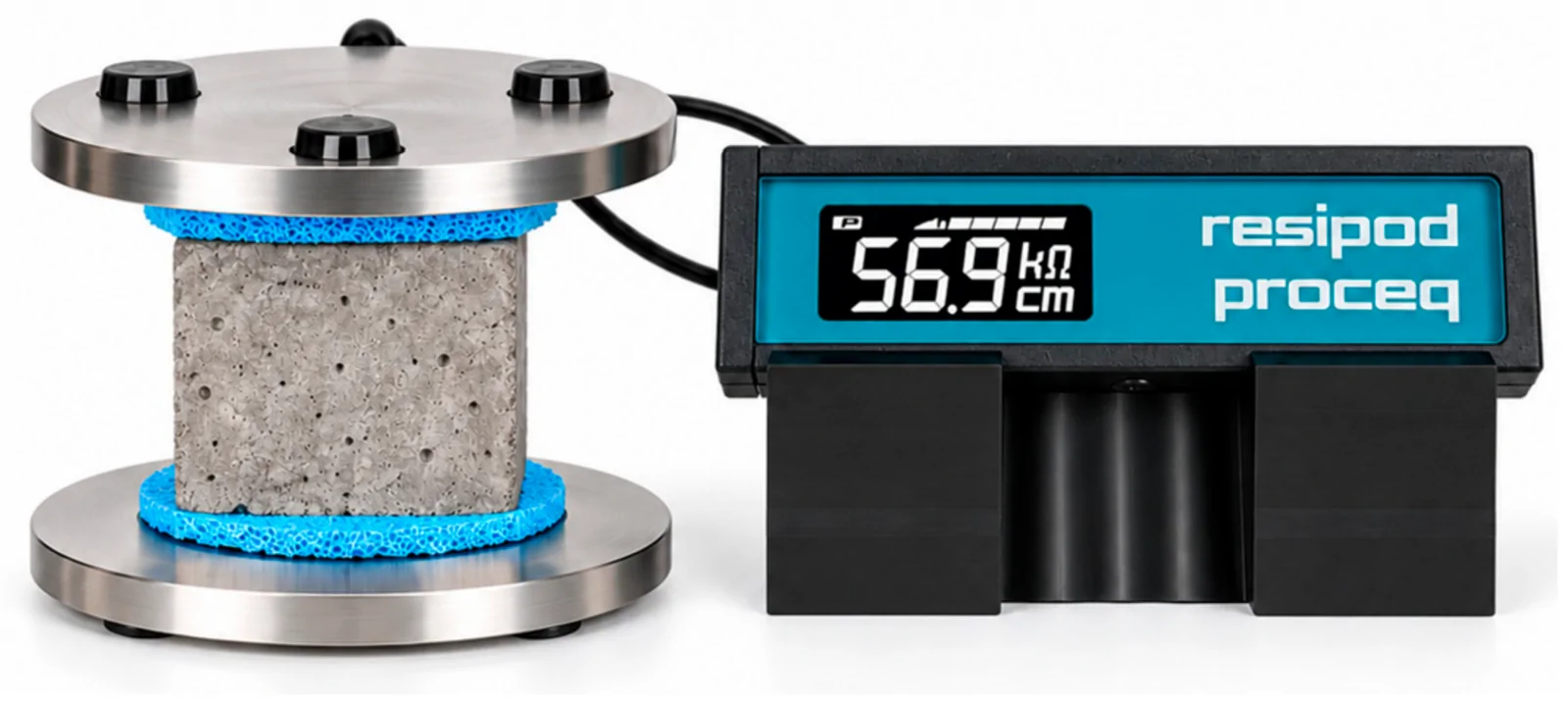
Bulk electrical resistivity test setup for 50 mm geopolymer mortar cubes using stainless-steel plate electrodes, saturated sponge contact layers, and a Resipod meter.

**Figure 10:**
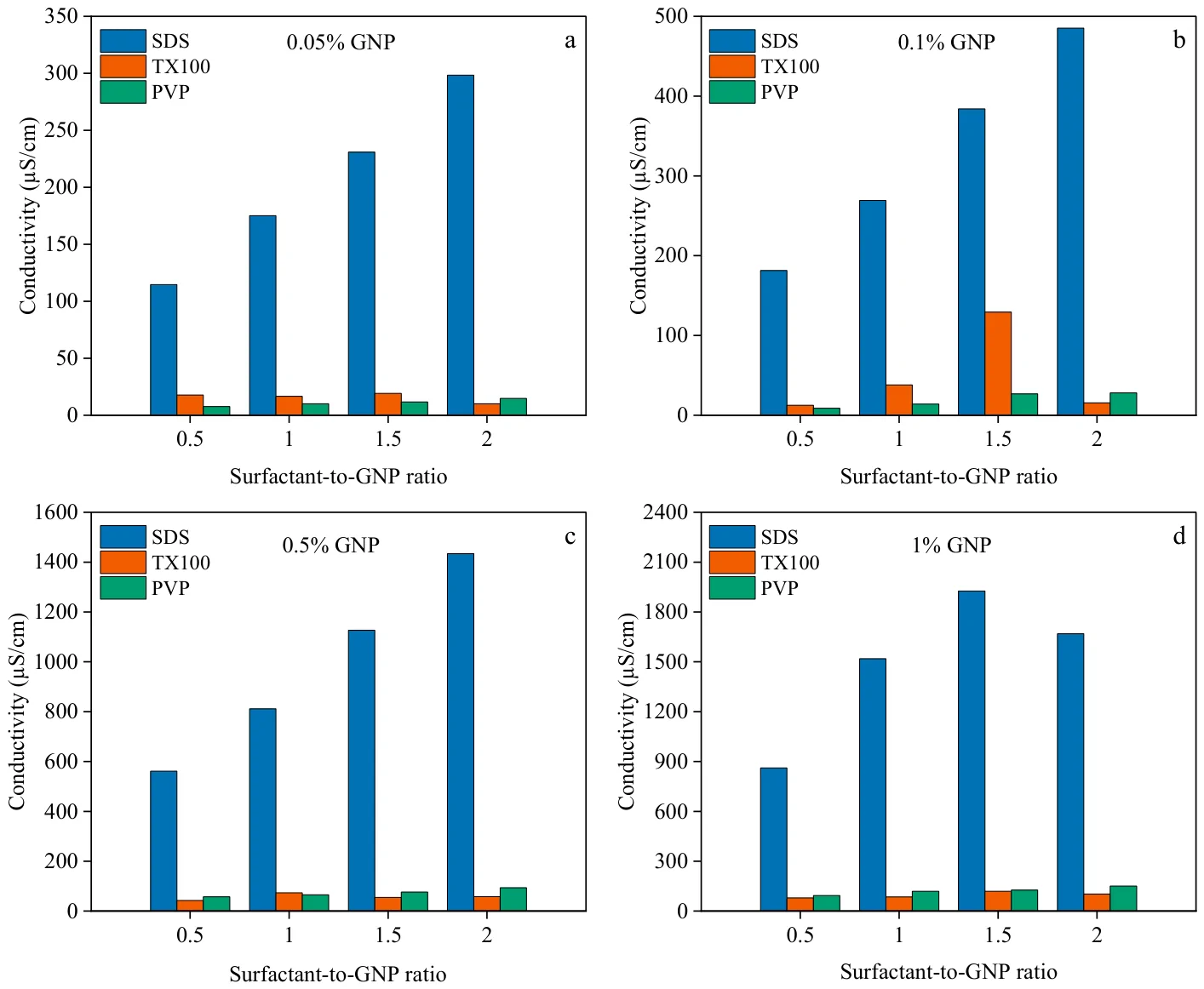
Electrical conductivity of aqueous GNP dispersions prepared with SDS, TX-100, and PVP at different GNP concentrations and surfactant-to-GNP mass ratios. (**a**) 0.05%% GNP; (**b**) 0.1% GNP; (**c**) 0.5% GNP; (**d**) 1% GNP.

**Figure 11:**
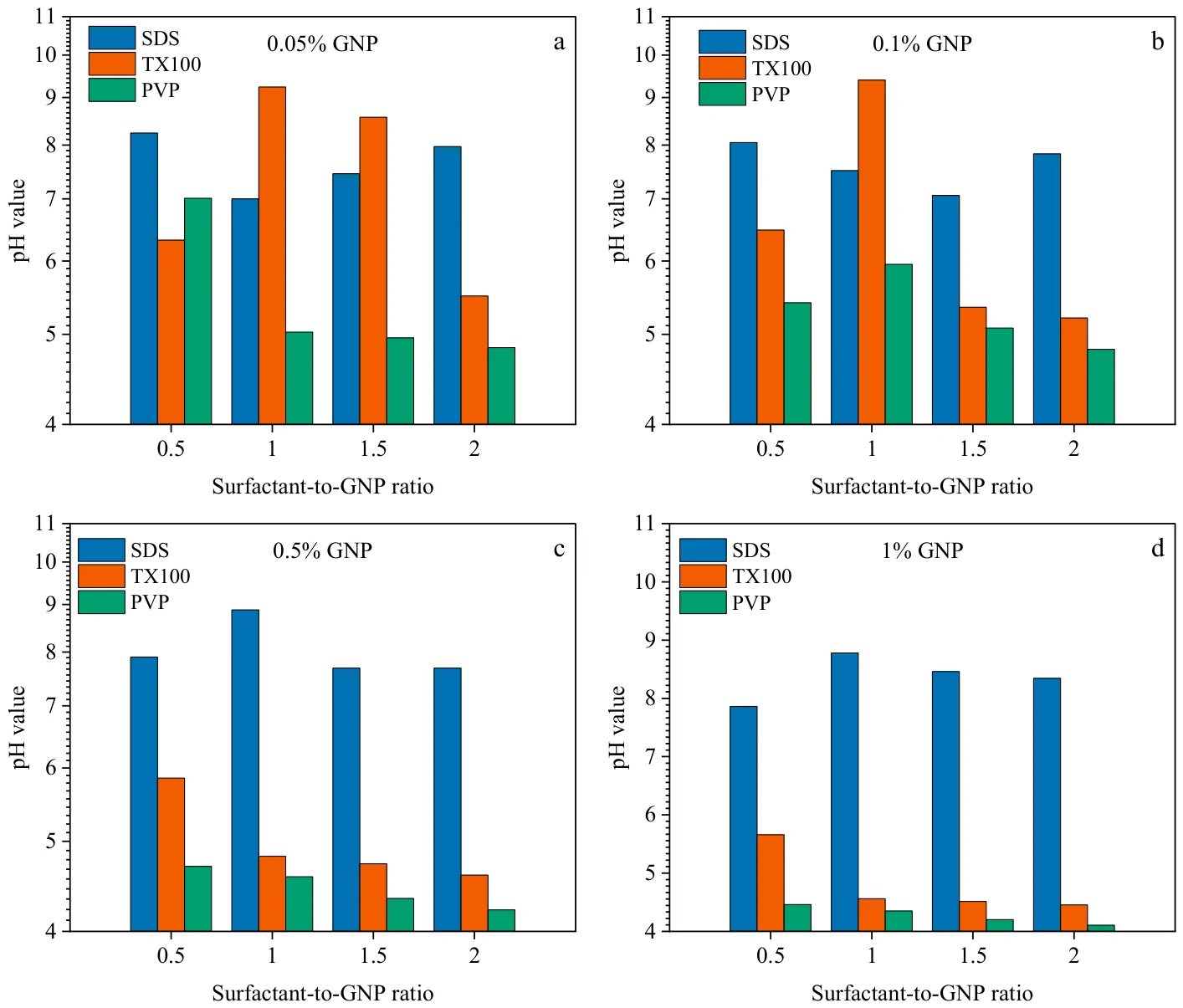
pH of GNP dispersions prepared with SDS, TX-100, and PVP at different GNP concentrations and surfactant-to-GNP mass ratios. (**a**) 0.05%% GNP; (**b**) 0.1% GNP; (**c**) 0.5% GNP; (**d**) 1% GNP.

**Figure 12:**
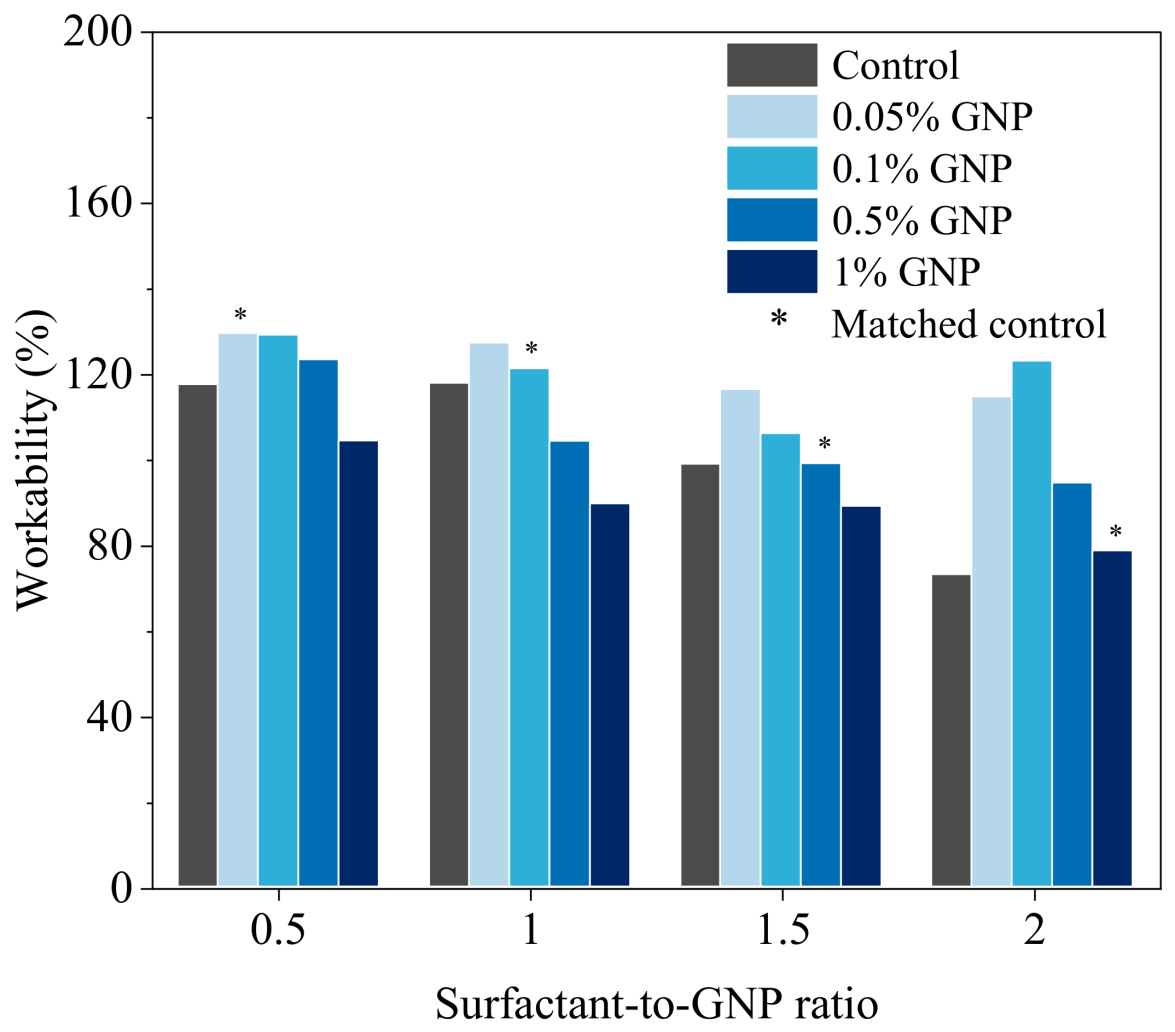
Flow table results of geopolymer mortar mixtures made with different GNP concentrations and surfactant-to-GNP mass ratios. Asterisks indicate GNP-containing mixtures with SDS content matched to the corresponding control.

**Figure 13:**
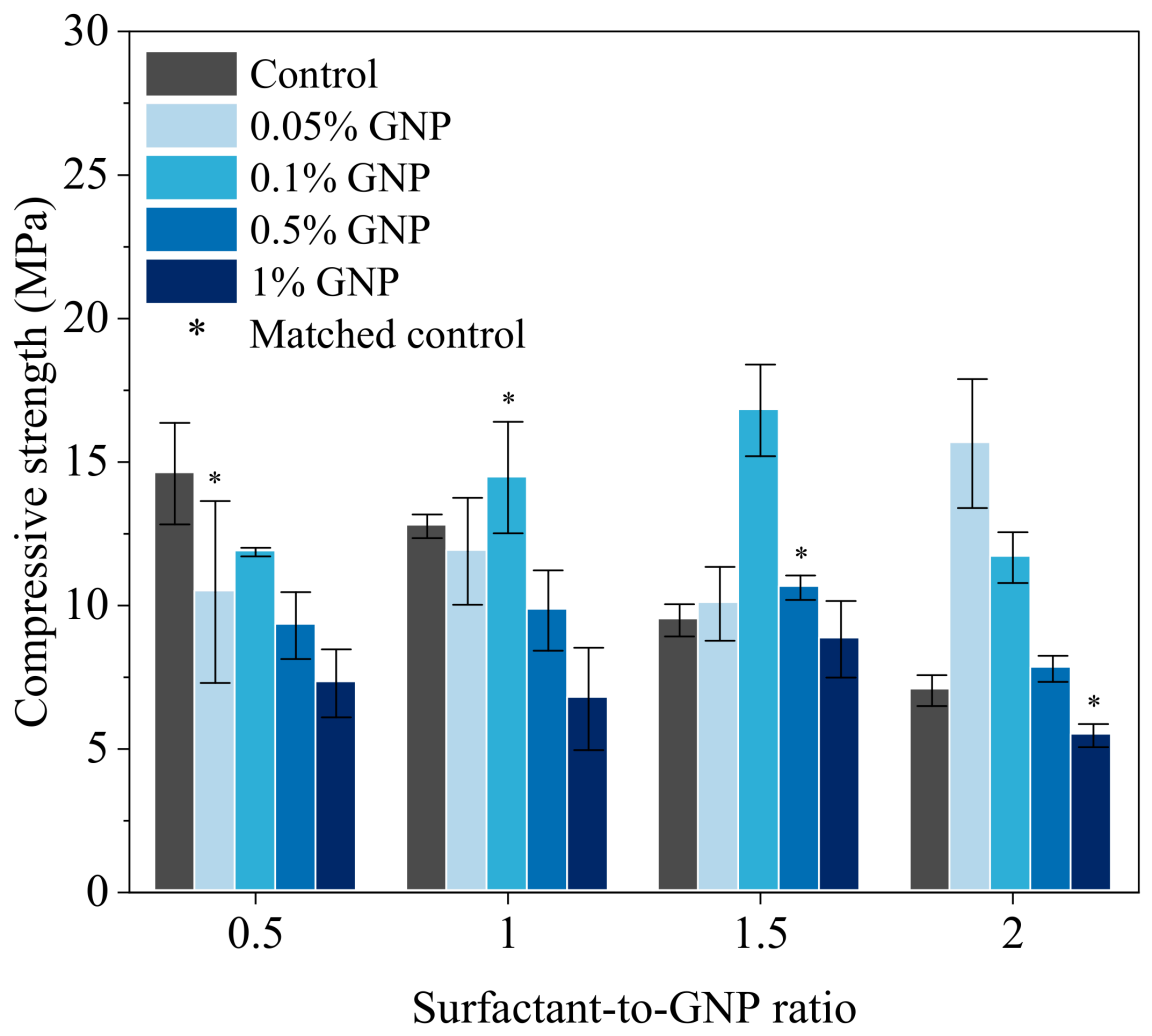
Compressive strength results of 28 d geopolymer mortar specimens made with different GNP concentrations and surfactant-to-GNP mass ratios. Error bars represent 95% confidence intervals for the mean (*n* = 3). Asterisks indicate GNP-containing mixtures with SDS content matched to the corresponding control.

**Figure 14:**
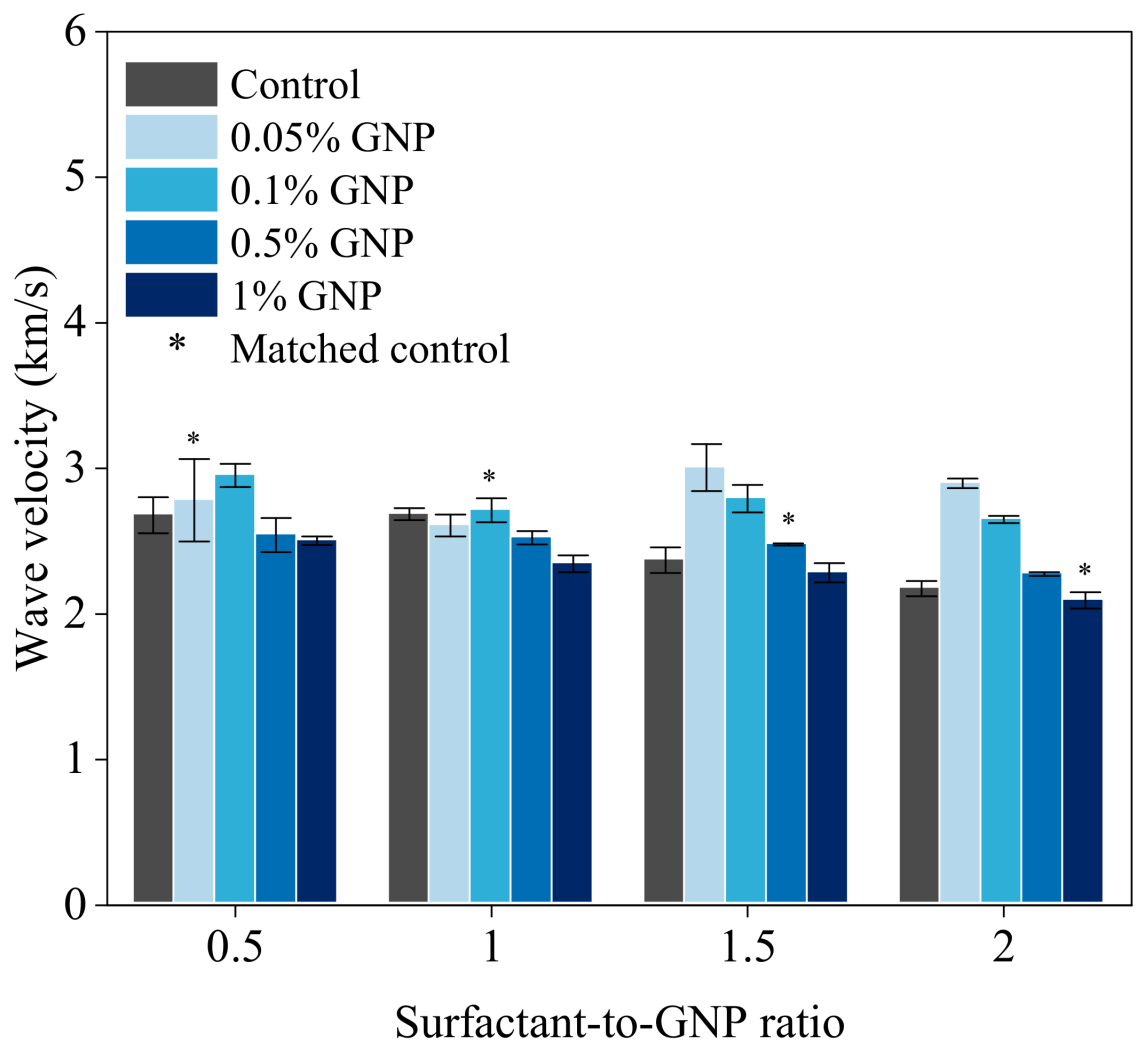
UPV results of 28 d geopolymer mortar specimens made with different GNP concentrations and surfactant-to-GNP mass ratios. Error bars represent 95% confidence intervals for the mean (*n* = 3). Asterisks indicate GNP-containing mixtures with SDS content matched to the corresponding control.

**Figure 15:**
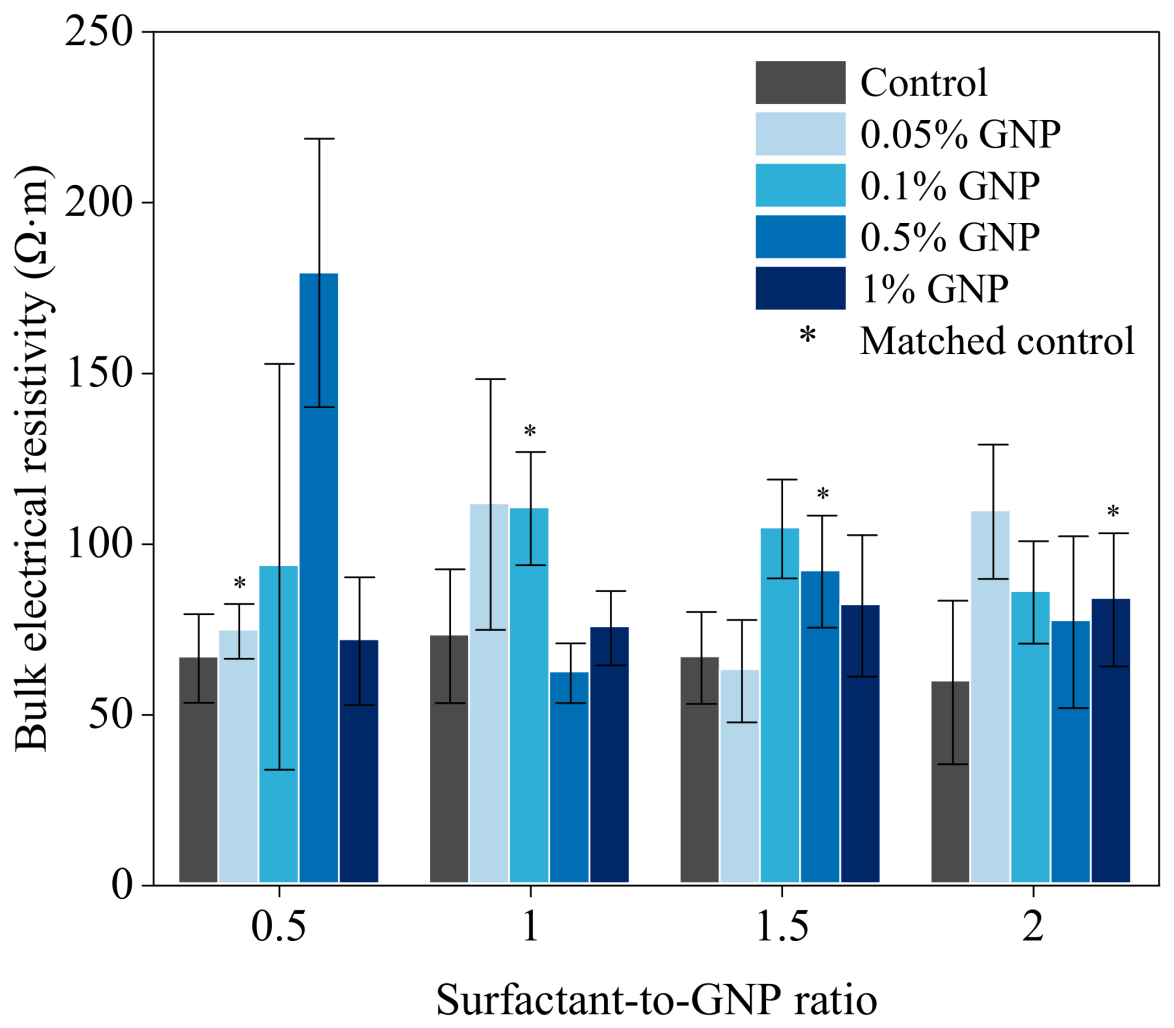
Bulk electrical resistivity results of 28 d as-cured geopolymer mortar specimens made with different GNP concentrations and surfactant-to-GNP mass ratios (*n* = 3). Asterisks indicate GNP-containing mixtures with SDS content matched to the corresponding control.

**Figure 16:**
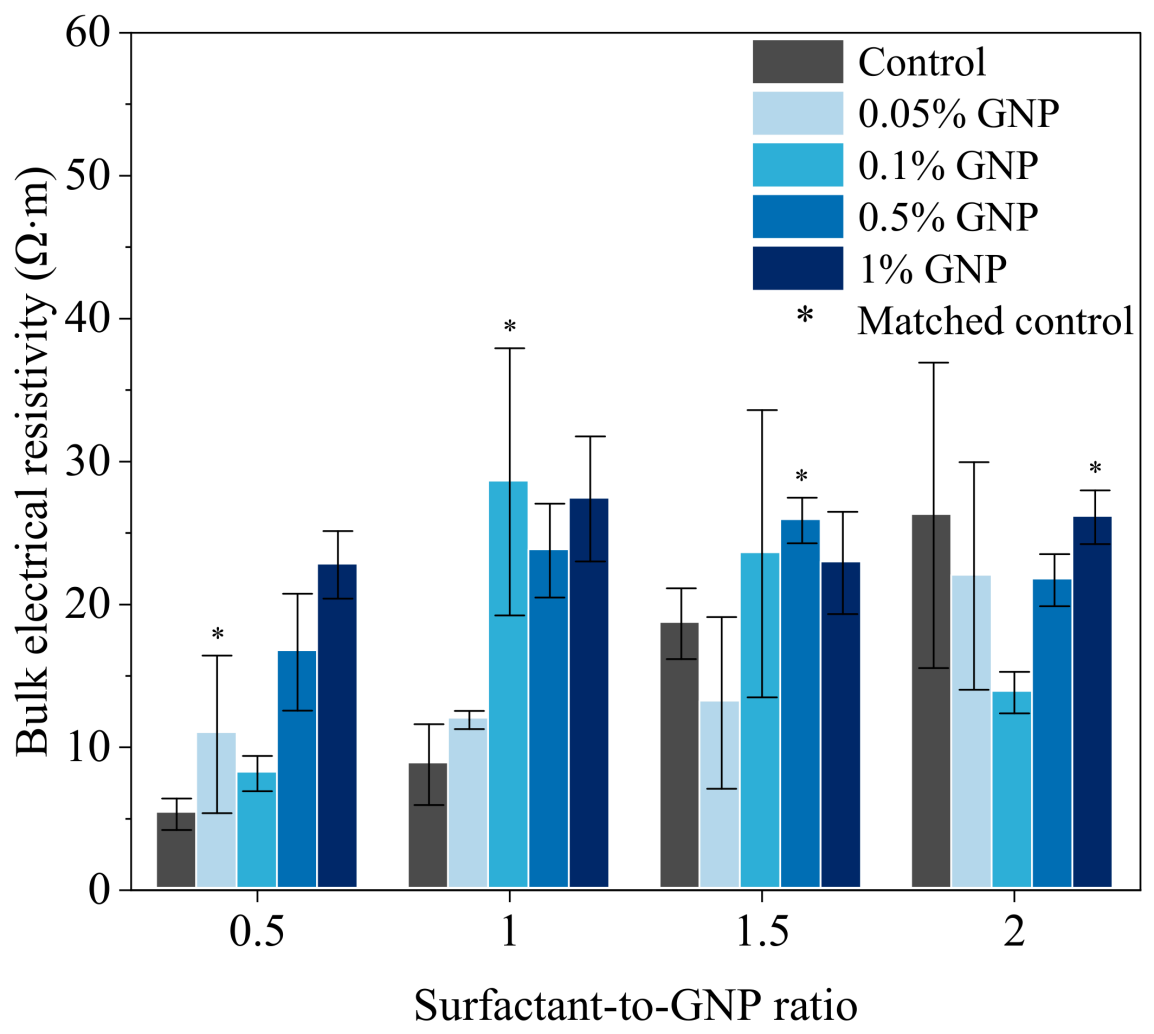
Bulk electrical resistivity results of 28 d, 24 h water-immersed, surface-dried geopolymer mortar specimens made with different GNP concentrations and surfactant-to-GNP mass ratios. Error bars represent 95% confidence intervals for the mean (*n* = 3). Asterisks indicate GNP-containing mixtures with SDS content matched to the corresponding control.

**Figure 17:**
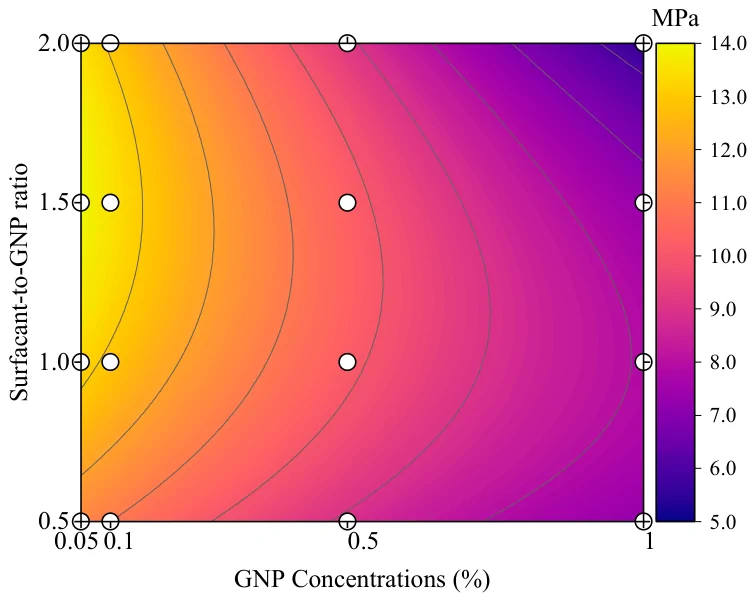
Response surface heat map for fitted compressive strength as a function of GNP concentration and surfactant-to-GNP ratio. Open circles indicate the experimental design points, while the contour lines represent combinations of factor levels yielding the same model-predicted response. Open circles indicate the experimental design points used to fit the response-surface model.

**Figure 18:**
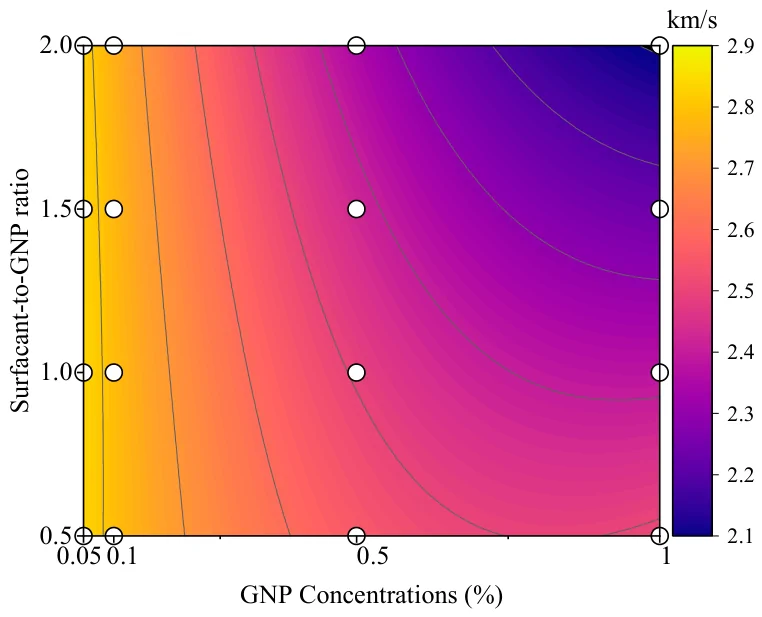
Response surface heat map for fitted UPV as a function of GNP concentration and surfactant-to-GNP ratio. Open circles indicate the experimental design points, while the contour lines represent combinations of factor levels yielding the same model-predicted response.

**Figure 19:**
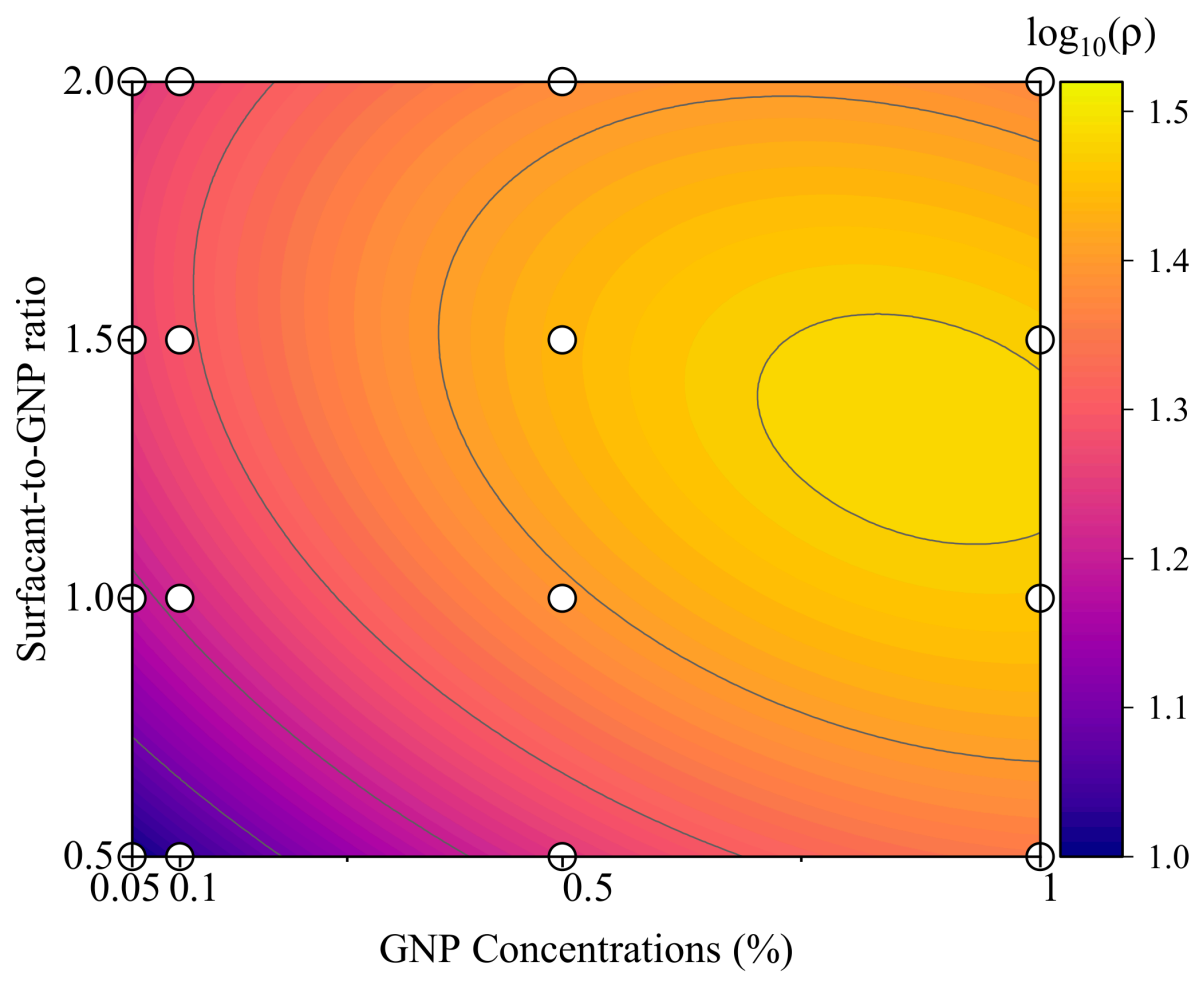
Response surface heat map for fitted log-transformed bulk electrical resistivity as a function of GNP concentration and surfactant-to-GNP ratio. Open circles indicate the experimental design points, while the contour lines represent combinations of factor levels yielding the same model-predicted response.

**Figure 20:**
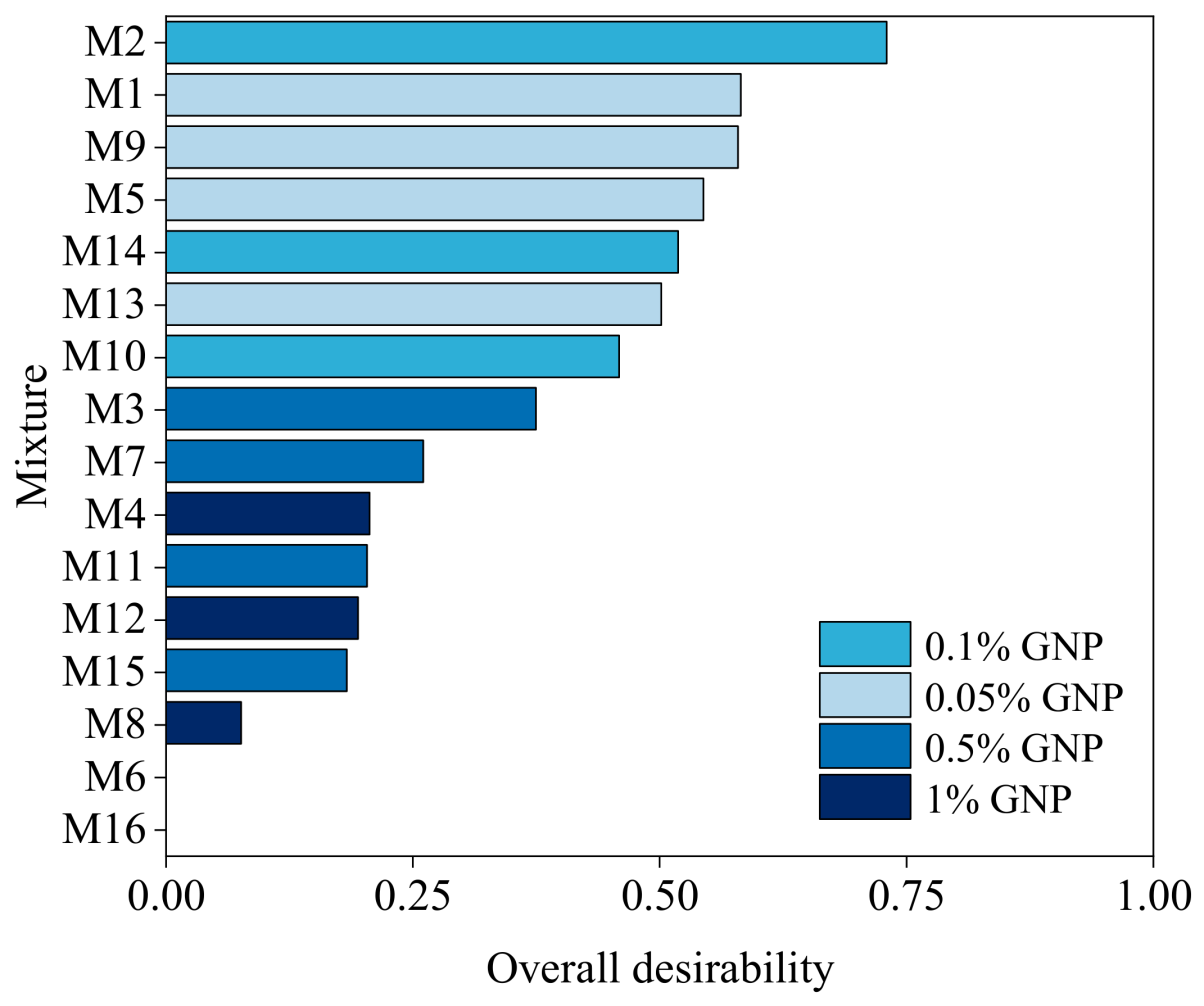
Desirability ranking of GNP-containing geopolymer mortar mixtures.

**Table 1:** Oxide composition and loss on ignition (LOI) of Class F fly ash (%mass).

SiO_2_	Al_2_O_3_	Fe_2_O_3_	CaO	MgO	SO_3_	Na_2_O	K_2_O	LOI
54.24	18.16	10.42	7.37	1.46	1.30	1.24	2.71	1.13

**Table 2:** Physicochemical properties of surfactants used for GNP dispersion.

Surfactant	pH	Density (g/cm^3^)	Purity (%)
TX-100	9.7	1.07	90 to 100
PVP	5.0	1.69	99
SDS	9.1	0.370	98.40

**Table 3:** Mass of surfactant used per 100 g water in the dispersion screening design.

GNP per 100 g Water (g)	Surfactant Mass in Grams			
	0.5:1 Ratio	1:1 Ratio	1.5:1 Ratio	2:1 Ratio
0.05	0.025	0.05	0.075	0.10
0.10	0.05	0.10	0.150	0.20
0.50	0.25	0.50	0.750	1.00
1.00	0.50	1.00	1.50	2.00

**Table 4:** Mixture proportions of geopolymer mortar, with GNP dosage calculated from the listed GNP mass divided by 300 g fly ash. Na_2_SiO_3_ denotes the commercial sodium silicate solution, not anhydrous sodium metasilicate.

ID	Fly Ash (g)	Sand (g)	Na_2_SiO_3_ (g)	NaOH (g)	GNP Dispersion Solution (g)	GNPs (g)	Surfactant (g)	Extra Water (g)	GNP Dosage (%wt. Fly Ash)
M1	300	600	124.5	24.5	25.6	0.0128	0.006	10.4	0.0042
M2	300	600	124.5	24.5	25.6	0.0256	0.013	10.4	0.0085
M3	300	600	124.5	24.5	25.6	0.1278	0.064	10.4	0.0426
M4	300	600	124.5	24.5	25.6	0.2556	0.128	10.4	0.0852
M5	300	600	124.5	24.5	25.6	0.0128	0.013	10.4	0.0042
M6	300	600	124.5	24.5	25.6	0.0256	0.026	10.4	0.0085
M7	300	600	124.5	24.5	25.6	0.1278	0.128	10.4	0.0426
M8	300	600	124.5	24.5	25.6	0.2556	0.256	10.4	0.0852
M9	300	600	124.5	24.5	25.6	0.0128	0.019	10.4	0.0042
M10	300	600	124.5	24.5	25.6	0.0256	0.038	10.4	0.0085
M11	300	600	124.5	24.5	25.6	0.1278	0.192	10.4	0.0426
M12	300	600	124.5	24.5	25.6	0.2556	0.383	10.4	0.0852
M13	300	600	124.5	24.5	25.6	0.0128	0.026	10.4	0.0042
M14	300	600	124.5	24.5	25.6	0.0256	0.051	10.4	0.0085
M15	300	600	124.5	24.5	25.6	0.1278	0.256	10.4	0.0426
M16	300	600	124.5	24.5	25.6	0.2556	0.511	10.4	0.0852
C 1	300	600	124.5	24.5	25.6	0	0.006	10.4	0
C 2	300	600	124.5	24.5	25.6	0	0.026	10.4	0
C 3	300	600	124.5	24.5	25.6	0	0.192	10.4	0
C 4	300	600	124.5	24.5	25.6	0	0.511	10.4	0

**Table 5:** Response surface regression coefficients for strength, UPV, and log-transformed resistivity.

Response	*b*_0_	*b*_1_	*b*_2_	*b*_11_	*b*_22_	*b*_12_	*R* ^2^	RMSE
Strength	10.951 ***	−2.624 ***	0.237	0.361	−0.776 †	−0.587 †	0.622	1.996
UPV	2.514 ***	−0.259 ***	−0.063 **	0.082 **	-0.004	−0.063 **	0.791	0.120
log_10_(*ρ*)	1.380 ***	0.125 ***	0.059 **	−0.05	−0.067 *	−0.033 †	0.528	0.125

Note: The fitted model was $Y=b_{0}+b_{1}G_{c}+b_{2}R_{c}+b_{11}G_{c}^{2}+b_{22}R_{c}^{2}+b_{12}G_{c}R_{c}+\varepsilon$, where $G_{c}$ is coded GNP concentration, $R_{c}$ is coded surfactant-to-GNP ratio, and ε is the residual error. Statistical significance: *** *p* < 0.001, ** *p* < 0.01, * *p* < 0.05, † 0.05 ≤ *p* < 0.10.

## Data Availability

The original contributions presented in this study are included in the article. Further inquiries can be directed to the corresponding author.
